# Engineered plant-derived extracellular vesicles for targeted regulation and treatment of colitis-associated inflammation

**DOI:** 10.7150/thno.97139

**Published:** 2024-09-03

**Authors:** Su Jin Kang, Jeong Hyun Lee, Won Jong Rhee

**Affiliations:** 1 Department of Bioengineering and Nano-Bioengineering, Incheon National University Incheon 22012, Republic of Korea.; 2 Division of Bioengineering, Incheon National University Incheon 22012, Republic of Korea.; 3 Research Center for Bio Materials & Process Development, Incheon National University, Incheon 22012, Republic of Korea.

**Keywords:** Inflammatory bowel diseases, Extracellular vesicles, Plant, Red cabbage-derived extracellular vesicles, t-Rabex

## Abstract

**Rationale:** Inflammatory bowel disease (IBD) is a chronic disorder characterized by persistent inflammation of the gastrointestinal tract. Due to the elusive causes and complex mechanisms of this disorder, the development of highly effective therapeutic drugs is crucial. Extracellular vesicles (EVs) are small membrane-bound structures released by cells into the surrounding environment. Recent research has witnessed a substantial surge in the utilization of plant-derived EVs that offer advantages such as high productivity, low production costs, diverse biological functions, and low cytotoxicity. Herein, Red cabbage-derived EVs (Rabex) were investigated and engineered as potential therapeutic agents for IBD.

**Methods:** Rabex was engineered by surface conjugation with hyaluronic acid (t-Rabex) to simultaneously enhance the targeting of intestinal epithelial and immune cells, thereby improving their therapeutic targeting and efficacy. The properties and therapeutic potential of t-Rabex were assessed through both *in vitro* studies and *in vivo* experiments, focusing on their capacity to reach the gastrointestinal tract and exert a therapeutic effect compared to unmodified Rabex.

**Results:** Rabex exhibited dual functions, including the suppression of inflammation in macrophages and promotion of colon epithelial cell regeneration, both of which are critical for effective IBD treatment. *In vitro* and *in vivo* studies of t-Rabex have demonstrated its superior targeting efficiency to the gastrointestinal tract and therapeutic efficacy compared to Rabex, making it a promising and more effective IBD treatment. Understanding the mechanism of action of t-Rabex in colonic tissues highlighted its anti-inflammatory, antioxidative, and tight-junction maintenance properties.

**Conclusions:** These findings underscore the potential of t-Rabex as a precise therapeutic agent for IBD and shed light on the diverse applications of plant-derived EVs.

## Introduction

Inflammatory bowel disease (IBD) is a chronic disorder characterized by persistent inflammation of the gastrointestinal tract and primarily includes ulcerative colitis (UC) and Crohn's disease (CD) 1-3. They are distinguished based on clinical features and differences. Ulcerative colitis primarily affects the colon, whereas Crohn's can cause inflammation anywhere in the gastrointestinal tract, often causing abdominal pain, weight loss, and various complications. Recent trends have revealed a notable increase in the incidence of IBD 4. The precise etiology of IBD remains elusive, although it is believed to be influenced by the complex interplay between environmental, genetic, and immune factors. Crohn's disease in particular is a severe and refractory form of IBD that is characterized by chronic and aggressive inflammation that can affect the entire digestive system from the mouth to the anus, ultimately leading to symptoms such as abdominal pain, diarrhea, bloody stools, and, in severe cases, fever, anemia, and significant weight loss 5-7. Although a range of treatments exist for IBD, including aminosalicylates, corticosteroids, immunomodulators, and monoclonal antibodies, many patients do not respond adequately or experience steroid resistance, thus highlighting the need for more effective and novel therapeutic approaches 8-11.

Extracellular vesicles (EVs) are small membrane-bound structures released by cells into the surrounding environment, with exosomes being the smallest subtype and ranging from 50 to 150 nm in size 12-15. They are known for their ability to encapsulate specific molecules, including proteins, nucleic acids, lipids, and carbohydrates, within a lipid bilayer to provide stable protection while facilitating the transfer of these molecules to other cells 16-19. Due to their prevalence in bodily fluids such as blood, urine, and saliva, extracellular vesicles have emerged as promising biological entities with various potential applications 20-25. EVs are produced by various biological entities, including humans, animals, insects, microorganisms, and plants 26-28. Among them, EVs derived from plants that are natural substances have recently been highlighted due to several advantages over EVs produced from cell cultures, including functional diversity among plant species and minimal concerns regarding side effects 29-36. Moreover, plant-derived EVs can be directly isolated from plants, thus ensuring high productivity without using a scaled-up bioreactor operation that requires high costs and complex techniques. Despite these advantages, comprehensive studies examining the production processes, biological functions, and therapeutic applications of plant-derived extracellular vesicles remain unexplored.

**Scheme 1:**
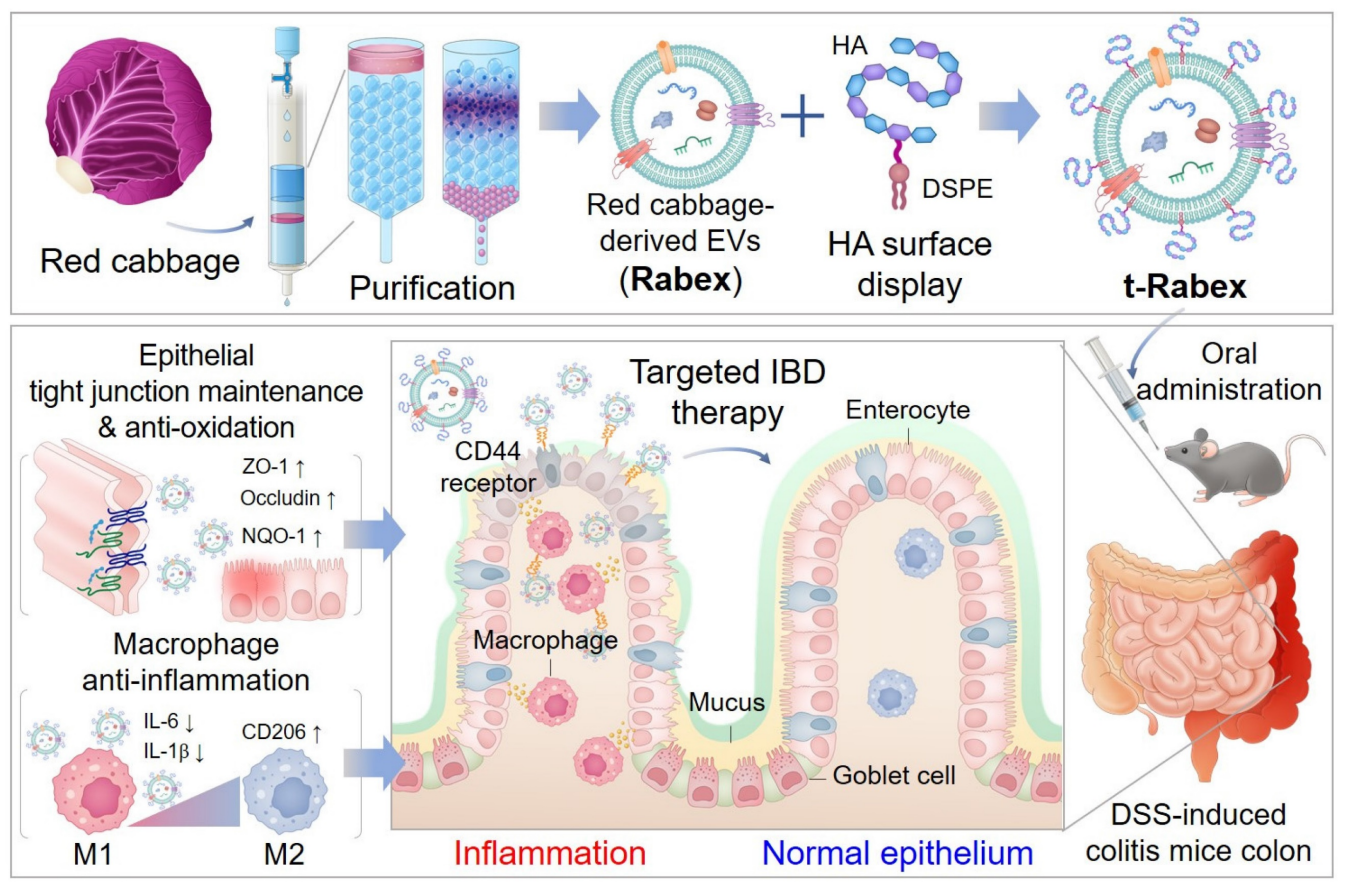
Development of red cabbage-derived EVs (Rabex) and surface engineered Rabex (t-Rabex) for the efficient and targeted IBD therapy.

In our previous work, large amounts of EVs were isolated from red cabbage (*Brassica oleracea*), denoted as Rabex, using ultrafiltration and size exclusion chromatography, and they were further evaluated for their biological functions, including the promotion of proliferation and inhibition of inflammation 37. In this study, Rabex was comprehensively investigated as a therapeutic agent for IBD in *in vivo* and *in vitro* models (Scheme 1). Oral administration of Rabex in dextran sulfate sodium (DSS)-treated mice successfully improved the inflamed intestinal areas of the colon and significantly decreased disease activity index (DAI) scores. Furthermore, to maximize the therapeutic efficacy of Rabex and thereby treat severe IBD, Rabex was engineered on its surface with hyaluronic acid (HA), a ligand for CD44, to provide enhanced targeted therapeutic effects for IBD. We demonstrated that the surface display of Rabex with HA (t-Rabex) increased the delivery of Rabex to both the immune and epithelial cells, thereby increasing its therapeutic efficacy in a Rabex *in vivo* mouse model. These results suggest that our strategy may offer an alternative and replaceable immunotherapy for IBD using plant-derived EVs and their engineered derivatives. This represents a significant step toward more effective and targeted therapies in the field of IBD treatment and paves the way for further research and clinical applications.

## Materials and methods

### Animals

Male C57BL/6 mice aged 6 - 8 weeks were procured from NARA Biotech. These mice were housed under controlled environmental conditions, including temperature and humidity, with a 12 h light-dark cycle. All experiments were conducted in compliance with the regulations and ethical guidelines of the Incheon National University Animal Experiment Ethics Committee and were approved (INU-ANIM-2022-12).

### Cell culture

Human colon cancer-derived intestinal epithelial cells (Caco-2) and human monocytes (THP-1) were cultured in Minimum Essential Medium Eagle (MEM), RPMI-1640 (Corning, USA), supplemented with 10% (v/v) fetal bovine serum (FBS, Gibco, MA, USA), and 1% (v/v) penicillin and streptomycin (Gibco, MA, USA). All cell lines were incubated in a humidified atmosphere of 5% CO_2_ at 37°C. Inflammation was induced using lipopolysaccharide (LPS; Sigma-Aldrich, USA, MO, USA) and DSS (MB Biomedicals, CA, USA).

### Isolation of extracellular vesicles from red cabbage

Rabex vesicles were extracted from red cabbage (*Brassica oleracea* var.* capitata* F.* rubra*) obtained from local Korean farms through a process of blending, centrifugation, ultrafiltration, and size exclusion chromatography (Izon Science, New Zealand).

### NTA, TEM, and zeta potential analysis

The size distribution and concentration of Rabex were analyzed using a Nanosight NS300 (Malvern, UK). For TEM, vesicles were absorbed onto a Formvar carbon-coated grid for 10 min. After washing the grid with distilled water, Rabex were fixed with 2% paraformaldehyde and washed twice with PBS. The grids were negatively stained with 2% uranyl acetate for 10 min. The samples were dried for 15 min and visualized using a JEM-1010 electron microscope (JEOL, Japan). The zeta potential and PDI of Rabex were assessed using Zetasizer Nano ZS (Malvern, UK).

### Confocal microscopy of Rabex uptake into cells

Rabex uptake by cells was assessed using a confocal microscope (Zeiss, Germany). Vesicles were stained with PKH67 dye (Sigma-Aldrich), incubated with cells, and analyzed after nuclear staining with Hoechst 33342 (Sigma-Aldrich).

### Flow cytometry analysis of Rabex intracellular delivery mechanism using endocytosis inhibitors

To confirm the intracellular delivery mechanism of Rabex, Caco-2 cells were treated with endocytosis inhibitors for 1 h at 37°C, followed by treatment with PKH-labeled Rabex for 4 h. Flow cytometry was used for analysis (Beckman Coulter, Inc., CA, USA). Clathrin-mediated endocytosis (CME) was inhibited with 30 µM chlorpromazine, phagocytosis with 1 µM cytochalasin D, macropinocytosis with 200 µM amiloride, and caveolin-mediated endocytosis with 7.5 µM filipin complex. The results were expressed as the percentage of Rabex uptake relative to the CTRL.

### DSS-induced mouse colitis model

Mice were administered 3% DSS (36,000 - 50,000 Da, MP Biomedicals) in their drinking water for a duration of 5 days, and this was followed by return to normal water. The CTRL mice received normal water throughout the experiment. Additionally, the oral administration of SSZ (100 mg/kg, Sigma-Aldrich), Rabex^LD^, Rabex^HD^, and t-Rabex^LD^ included one dose before DSS administration, five doses during the 5-day DSS treatment period, and four additional doses after DSS administration for a total of 10 doses over a 12-day period. Daily changes in body weight were monitored throughout the 14-day experimental period. At the conclusion of the experiment, the mice were euthanized, and their colons and organs were harvested. We measured colon length and sectioned pieces from the distal part for histological examination and immunofluorescence staining. We obtained 0.5 cm of the proximal colon tissue near the cecum and 0.5 cm of the distal colon tissue near the rectum, each of which was used for qRT-PCR analysis. The DAI and histological scores were measured over a 14-day period following the criteria outlined in Table S1 and Table S2. To assess biocompatibility, organs including the lungs, heart, liver, spleen, and kidneys were harvested and subjected to H&E staining (Dako, CA, USA). The stained tissues were analyzed using an optical microscope (Leica, Germany).

### *In vitro* digestion assay

To assess the stability of Rabex in the GI tract, an *in vitro* digestion test was conducted. For simulating gastric fluids, 1 mL of Rabex was incubated with 1.34 μL of 18.5% w/v HCl (pH 2.0) and 24 µL of pepsin (80 mg/mL in 0.1 M HCl) at 37°C for 1 h. For intestinal fluid simulation, 80 μL of a pancreatin (4 mg/mL) and bile extract (24 mg/mL) mixture in 0.1 M NaHCO_3_ was added while adjusting the pH to 6.5 using 1 M NaHCO_3_. The mixture was then incubated for 1 h under the same conditions. After digestion, the suspensions were cooled to 4°C to inactivate the enzymes.

### Effects of Rabex on cell viability, proliferation, and inflammation

To measure cell viability and proliferation, cells were seeded into 24-well plates and incubated for 24 h. Cells were incubated with EV-depleted FBS-containing media in the presence or absence of Rabex at concentrations ranging from 1.0 × 10^8^ to 1.0 × 10^11^ particles/mL for 96 h. Cell viability and proliferation were measured using the WST-1 assay. THP-1 cells were differentiated into M0 macrophages using 200 ng/mL PMA and were then treated with Rabex at a concentration of 1 × 10^9^ to 1 × 10^11^ particles/mL. One day later, inflammation was induced using 250 ng/mL LPS. After 24 h, RNA was extracted to measure mRNA levels of CD163, TNF-α, and IL-1β. Furthermore, to assess the extent of oxidative stress, THP-1 cells were exposed to LPS and Caco-2 cells were exposed to DSS, and this was followed by measurement of ROS using DCF-DA (Sigma, MO, USA) staining and fluorescence microscopy. DSS-induced cell death was evaluated using a trypan blue assay to calculate cell viability. The TEER assay was performed using an EVOM^2^ (WPI, France).

### mRNA sequencing

To investigate the effect of Rabex on DSS-induced colitis, we performed mRNA sequencing of colon tissues from treated mice (MACROGEN, Korea). Following RNA extraction and quality control, sequencing was performed on a high-throughput platform. Data were normalized and analyzed for differential expression with a p-value cutoff of <0.05. The reproducibility was assessed using Pearson's correlation coefficients. Hierarchical clustering was applied to visualize gene expression patterns. Gene Ontology (GO) and Kyoto Encyclopedia of Genes and Genomes (KEGG) pathway analyses were conducted to identify significant biological pathways and functions of differentially expressed genes.

### Construction of t-Rabex

First, we synthesized a DSPE-PEG-HA conjugate to modify the surface of Rabex using HA. Conjugation was achieved by reacting the amine group of 1,2-DSPE-PEG-NH_2_ with the carboxylic group of HA using EDC/NHS chemistry. HA (30,000-50,000 Da, Glentham Life Sciences, UK) was dissolved in 0.5 mL buffer at a concentration of 5 mg. Next, 1-(3-Dimethylaminopropyl)-3-ethylcarbodiimide (EDC, Thermo Scientific, MA, USA) and N-hydroxysulfosuccinimide sodium salt (sulfo-NHS, Thermo Scientific, MA, USA) were added to the solution at a 1:1 molar ratio in MES buffer and allowed to react for 15 min. Subsequently, DSPE-PEG_2000_-NH_2_ (Biopharma PEG, MA, USA) was mixed at a concentration of 5 mg/mL and reacted for 2.5 hours. The supernatant was then dialyzed in a dialysis tube (Spectra/Por® MWCO 2 kDa, Fisher Scientific, MA, USA) against ultrapure water for 3 days. Finally, the product was lyophilized. Conjugation was confirmed using ^1^H-NMR (JEOL, Japan) and FTIR spectroscopy (Bruker, Billerica, MA, USA). To engineer DSPE-PEG-HA on the surface of Rabex (t-Rabex), we conducted the reaction at 37°C for 4 h. Subsequently, unbound DSPE-PEG-HA was separated using ultrafiltration. The resulting t-Rabex was further characterized to confirm successful conjugation.

### Targeting effect assessment of t-Rabex

To verify the enhanced targeting function of t-Rabex, both Rabex and t-Rabex were labeled with PKH67 green dye and incubated with Caco-2 and THP-1 cells. After incubation, the cells were cultured in medium with or without Rabex or t-Rabex. The uptake of Rabex and t-Rabex by cells was quantitatively measured using a flow cytometer (Beckman Coulter, Inc., CA, USA) and qualitatively analyzed using fluorescence microscopy (Eclipse Ti2, Nikon, Japan). To block the CD44 receptor, THP-1 cells were treated with 0.5 µg/mL CD44 antibody (Cell Signaling Technology, MA, USA) for 2 h. The cells were washed twice with PBS, then incubated with PKH-labeled t-Rabex for 2 h. After incubation, the cells were washed twice with PBS and measured by flow cytometry.

### *In vivo* distribution of Rabex and t-Rabex

To evaluate the biodistribution of Rabex and t-Rabex, we performed *in vivo* imaging using an IVIS. Both Rabex and t-Rabex were labeled with Vybrant™ DiD cell-labeling solution (Invitrogen, MA, USA) for tracking purposes with minimal autofluorescence interference. The labeled particles were administered orally, and their distribution was assessed 24 h and 48 h post-administration. Fluorescence imaging was performed to capture ventral intensity and distribution in various colons and organs. Imaging and analysis were conducted using a VISQUE InVivo Smart-LF system (Vieworks, Korea) with a Cy 5.5 filter channel.

### qRT-PCR analysis

To assess the effect of Rabex on inflammation-related gene expression, qRT-PCR was performed using the StepOnePlus Real-Time PCR System (Applied Biosystems, USA). RNA was isolated using the FavorPrep™ Tri-RNA Reagent (FAVORGEN, Austria) according to the manufacturer's protocol. cDNA was synthesized using ReverTra Ace qPCR RT Master Mix (Toyobo, Japan), and RT-PCR was performed using THUNDERBIRD SYBR qPCR mix (Toyobo, Japan). The primer information is listed in Table S3.

### Enzyme-linked immunosorbent assay (ELISA) for cytokine detection

To assess the effect of Rabex on cytokine expression, ELISA was performed using whole colon tissue. The concentrations of IL-1β was quantified using an ELISA kit (R&D Systems, MN, USA), according to the manufacturer's protocol. A Varioskan^TM^ Flash was used to measure the absorbance (Thermo Scientific, USA).

### Hemolysis assay

To evaluate the blood compatibility of Rabex, fresh blood was collected from mice in EDTA-treated tubes (BD Biosciences, NJ, USA). Initially, 0.2 mL of whole blood was mixed with 4 mL PBS, and this was followed by centrifugation at 10,000 × g for 5 min. This step was repeated by washing with PBS to isolate red blood cells (RBCs). The isolated RBCs were resuspended in 2 mL of PBS. Subsequently, 0.2 mL of the RBC suspension was combined with 0.8 mL of the test sample. Phosphate buffered saline (PBS) was used for negative CTRL, and nuclease-free water was used for positive CTRL. Rabex and t-Rabex were then added, and the mixture was incubated at 37°C for 1 h. The mixture was centrifuged at 10,000 × g for 5 min. Finally, 100 µL of the supernatant was transferred to a 96-well plate, and absorbance was measured at 414 nm. The hemolysis rate was calculated as (sample absorbance - negative control absorbance) / (positive control absorbance - negative control absorbance) × 100.

### Statistical analysis

The results are expressed as mean ± standard deviation (SD), n ≥ 3. Data analysis was conducted using one-way and two-way Analysis of Variance (ANOVA), with a p-value of less than 0.05 denoting statistical significance. All statistical evaluations were derived from independent experiments and were performed using GraphPad Prism 7.0 software (GraphPad, La Jolla, CA, USA).

## Results and Discussion

### Comprehensive characterization and consistent quality assessment of Rabex

**Figure 1:**
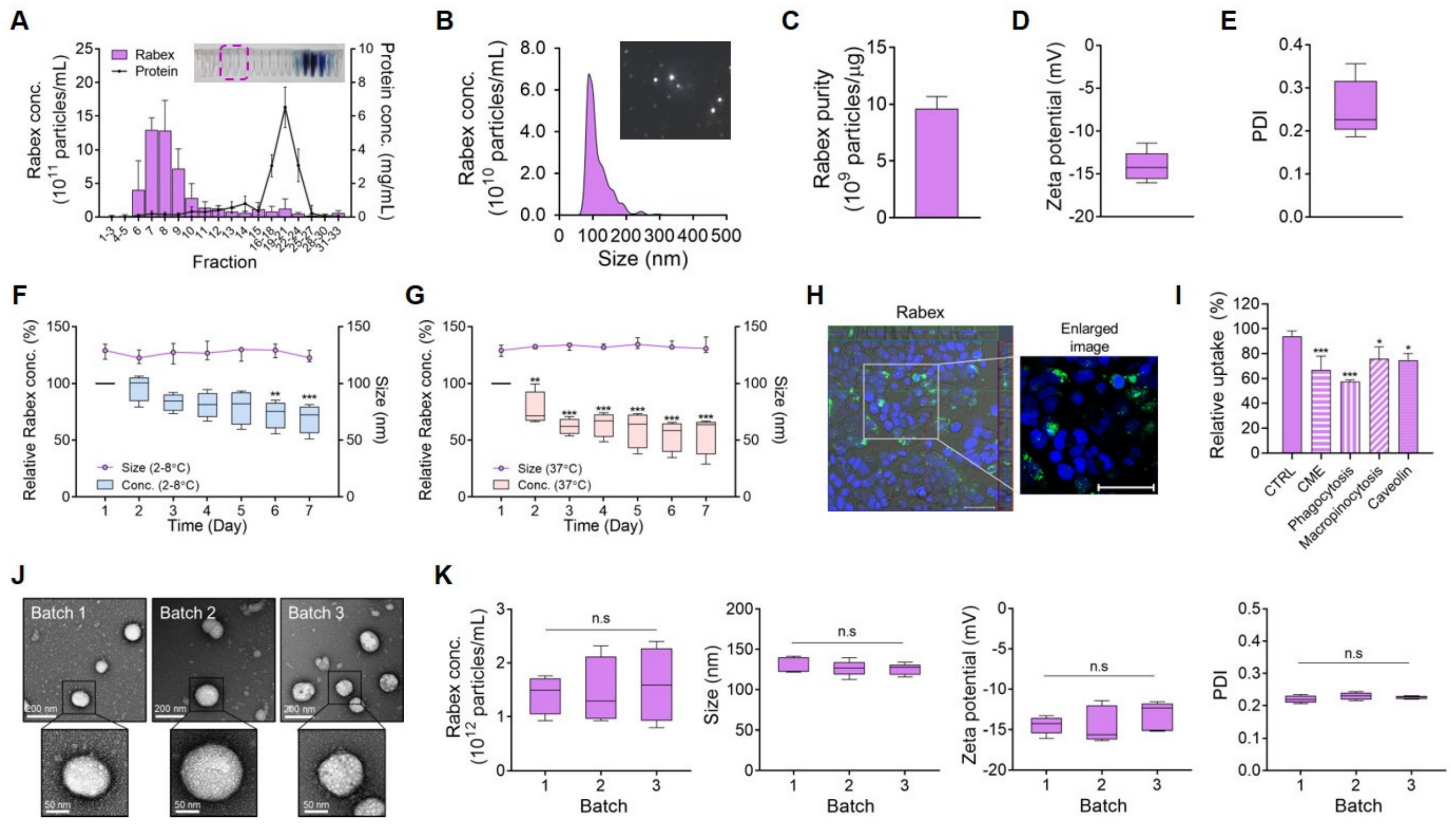
** Isolation and characterization of Rabex.** (**A**) EV fractional concentrations and protein impurity concentrations of Red cabbage juice after purification by size exclusion chromatography. EVs in the fraction 7 - 9 were further used as Rabex. (**B**) NTA for Rabex size and concentration assessments. (**C** - **E**) Purity (**C**), zeta potential (**D**), and PDI (**E**) were analyzed for Rabex. (**F** - **G**) Investigation of temperature- and time-dependent Rabex stability at 2 ─ 8°C (**F**) and 37°C (**G**) in PBS solution. (**H**) Confocal microscopy of Rabex intracellular delivery. Rabex was stained with PKH67 fluorescent dye and administered to Caco-2 cells. Size bars indicate 50 μm. (**I**) Flow cytometry analysis was performed to confirm the intracellular delivery mechanism of Rabex, using endocytosis inhibitors. Clathrin-mediated endocytosis (CME) was inhibited with chlorpromazine, phagocytosis with cytochalasin D, macropinocytosis with amiloride, and caveolin-mediated endocytosis with filipin complex. Results are expressed as the percentage of Rabex uptake relative to the CTRL. (**J**) TEM analysis of Rabex morphology among 3 different batches of Rabex, with size bars indicating 200 μm (upper panel) and 50 μm (lower panel), respectively. (**K**) Batch analysis of Rabex concentration, size, zeta potential, and PDI. The data are presented as mean ± SEM, n ≥ 3. Statistical significance is indicated as follows: *p < 0.05; **p < 0.01; ***p < 0.001; n.s, not significant.

Rabex in red cabbage juice was first concentrated using ultrafiltration, and this was followed by size exclusion chromatography to separate Rabex from contaminants, including proteins 37, 38. Size-exclusion chromatography revealed that the highest EVs particle concentrations were observed in fractions 7 and 9 (Figure 1A). Upon combining fractions 7-9 and performing nanoparticle tracking analysis (NTA), Rabex particles with a size of 115.2 nm were observed (Figure 1B). Evaluation of protein impurities demonstrated that Rabex was highly pure (Figure 1C). Additionally, Rabex exhibited a cost that was 97,721-fold lower than that of mammalian cell-derived extracellular vesicles when comparing the cost of mammalian cell-derived extracellular vesicles and other plant cell-derived extracellular vesicles (Figure S1). Further characterization using Zetasizer revealed properties of Rabex resembling typical EVs, with a zeta potential of -14.1 mV and a polydispersity index (PDI) value of 0.25 (Figure 1D- E).

To assess stability of Rabex, Rabex were incubated at temperatures of 2 - 8°C and 37°C in phosphate-buffered saline (PBS) while measuring the changes in size and relative concentration (Figure 1F-G). The size remained relatively stable until day seven, and the concentration consistently decreased under all conditions. To evaluate the stability of Rabex in the presence of serum, its concentration and size were assessed in 50% FBS (Figure S2A-B). The results revealed that Rabex remained relatively stable in the presence of serum regardless of temperature, thus indicating that the development of formulation for Rabex may further increase their stability during the storage (2 - 8°C) and clinical application (37°C).

To confirm the intracellular delivery of Rabex, Caco-2 colon epithelial cells were incubated with Rabex stained with PKH67 fluorescent dye and observed under a confocal microscope. As presented in Figure 1H, in contrast to the results of CTRL (Figure S3), Rabex successfully penetrated human cells, thus indicating that the active components of Rabex could be delivered into cells and participate in cell regulation. To further elucidate the intracellular delivery mechanism of Rabex, the uptake rates of Rabex were assessed using various endocytosis inhibitors. Caco-2 cells were treated with inhibitors for clathrin-mediated endocytosis (chlorpromazine), phagocytosis (cytochalasin D), macropinocytosis (amiloride), and caveolin-mediated endocytosis (filipin complex) to inhibit their respective pathways. Following the inhibitor treatments, cells were incubated with PKH67-labeled Rabex, and their uptake was analyzed using flow cytometry. The results confirmed that Rabex primarily utilizes clathrin-mediated endocytosis and phagocytosis pathways for intracellular delivery (Figure 1I).

One potential concern in using plant-derived EVs is the consistency of quality, as the chemical, physical, and biological properties of EVs can be affected by the plant cultivation environment, including weather. Considering that pharmaceutical drugs require consistent effects and behavior in the human body, it is very important to ensure that EV qualities do not vary among production conditions 39-41. To rule out this concern, three batches of Rabex (batches 1, 2, and 3) produced over a three-year period (with batches produced each year) were compared for their productivity and properties. Transmission electron microscopy (TEM) revealed the same spherical morphology of EVs among batches (Figure 1J). Furthermore, consistent results were obtained for all tested properties, including concentration, size, zeta potential, and PDI, across all batches (Figure 1K). The yield of Rabex for each batch was consistently observed at 1.13 × 10^10^, 1.13 × 10^10^, and 1.20 × 10^10^ particles/g, respectively, across all batches (Figure S4). Taken together, these results confirm the evaluation of Rabex quality consistency. Therefore, Rabex is suitable for developing pharmaceutical candidates for IBD treatment.

### Assessing the in vivo application of Rabex in a mouse colitis model and its impact on treating IBD

**Figure 2:**
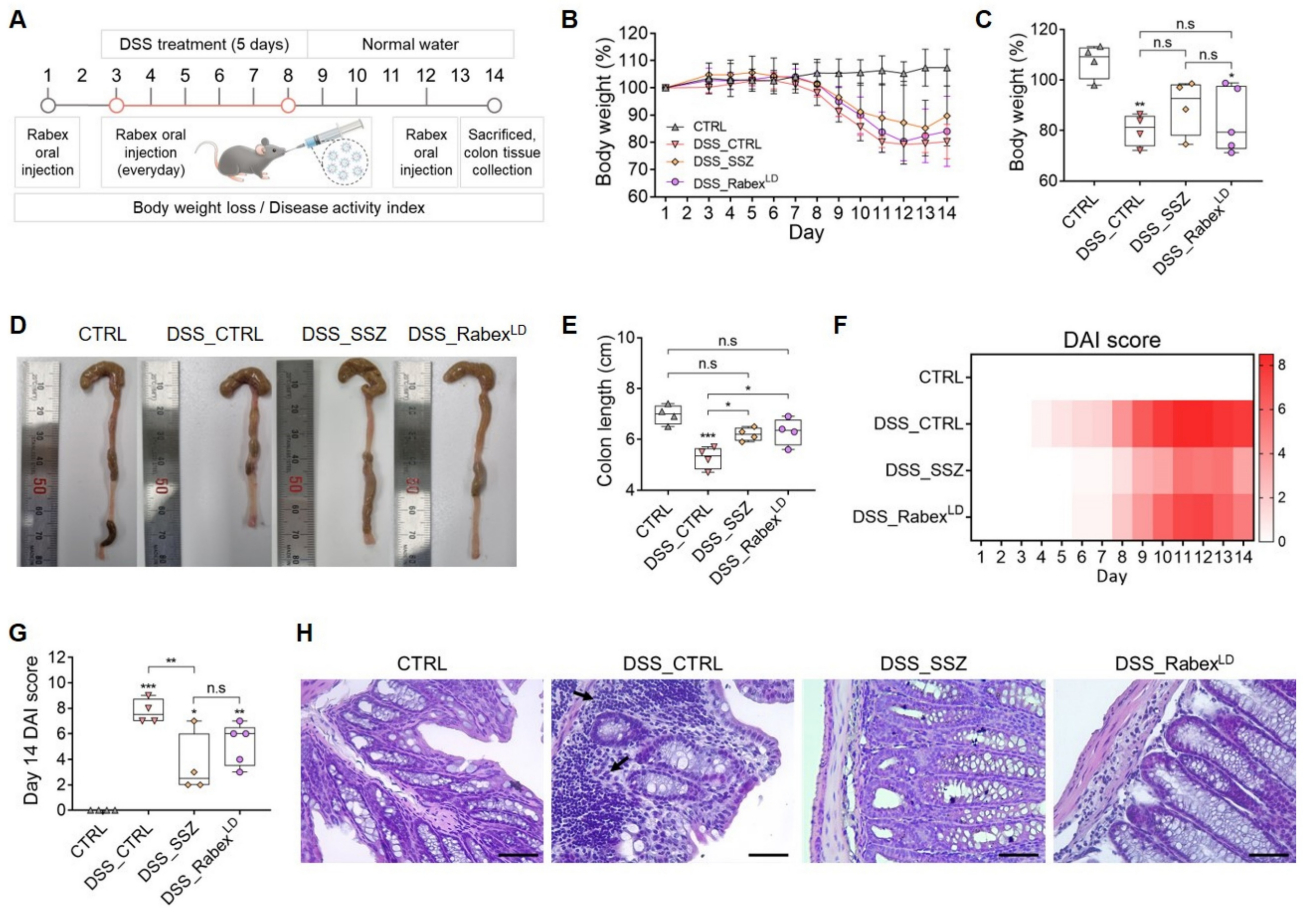
** Investigating the therapeutic efficacy of Rabex in an IBD mouse model.** (**A**) Schematic illustration of the development of a DSS-induced IBD model and the Rabex oral administration strategies. (**B**) Effect of Rabex administration on DSS-treatment-induced weight loss. SSZ was used as positive control drug. (**C**) The comparison of weight reduction on day 14 in the DSS_CTRL, SSZ (100mg/kg) and DSS_Rabex^LD^ (5 × 10^10^ particles/mL of Rabex) administered groups. (**D**, **E**) Representative photographs of isolated colons (**D**) and the comparison of colon length on day 14 resulting from IBD (**E**). Note that significant differences in colon length between DSS_CTRL, DSS_SSZ and DSS_ Rabex^LD^ was observed on day 14. (**F**, **G**) Heat map indicating DAI score change from day 1 to day 14. Significant differences in DAI scores between DSS_CTRL, DSS_SSZ and DSS_ Rabex^LD^ was observed on day 14. (**H**) Analysis of immune cell recruitment (black arrow) and the destruction of the intestinal epithelial layer confirmed through H&E staining, with size bars indicating 50 μm. The data are presented as mean ± SEM, n ≥ 3. Statistical significance is indicated as follows: *p < 0.05; **p < 0.01; ***p < 0.001; n.s, not significant.

In our previous study, Rabex was demonstrated to promote growth and inhibit the inflammatory response of macrophages 37. Therefore, we constructed a mouse model of DSS-induced colitis and orally administered Rabex to investigate its potential as a therapeutic candidate for IBD treatment (Figure 2A) 42-45. DSS induces colitis in mice by binding to medium-chain-length fatty acids in the colon and surface epithelium, ultimately leading to mucin loss and cell depletion 46. This in turn allows luminal microbes and their metabolites to enter the lamina propria to trigger an inflammatory response. Typical histological changes induced by acute DSS include mucin and cell depletion, epithelial erosion, and submucosal infiltration, ultimately resulting in increased immune cell infiltration despite an overall reduction in intestinal length 47-49.

To demonstrate that DSS administration leads to IBD development, the body weight, colon length, and DAI scores were measured and compared between the non-treated normal control (CTRL) and DSS-treated control (DSS_CTRL) groups. First, the body weight of the DSS_CTRL group was reduced to 19.8% of that of the CTRL group by day 14 (Figure 2B-C). Also, it was confirmed that DSS treatment caused an intestinal length decrease from 7.0 cm to 5.3 cm on average by day 14 (Figure 2D-E). DAI and histological scores were determined based on the predefined criteria outlined in Table S1 and Table S2, respectively. A time-dependent increase in the DAI score was observed in the DSS-treated group, where it reached 7.8 at day 14 after DSS treatment (Figure 2F-G). Thus, we confirmed the successful establishment of a DSS-induced colitis model.

Subsequently, to investigate its therapeutic effects, Rabex was orally administered at a concentration of 5 × 10^10^ particles/mL (hereafter referred to as Rabex^LD^). An experiment was conducted using sulfasalazine (SSZ, 100 mg/kg/day) as the positive control group. SSZ, composed of sulfapyridine and 5-aminosalicylic acid (5-ASA), is used as a treatment for IBD. The oral administration of SSZ and Rabex^LD^ included one dose before DSS administration, five doses during the 5-day DSS treatment period, and four additional doses after DSS administration for a total of 10 doses (Figure 2A). SSZ administration to DSS-treated mice (DSS_SSZ) resulted in a 10.4% reduction in body weight, and Rabex^LD^ administration to DSS-treated mice (DSS_Rabex^LD^) resulted in a 16.0% reduction in body weight. No significant difference was observed compared to the DSS_CTRL group (Figure 2B-C). However, it is noteworthy that the DSS_SSZ resulted in a colon length of 6.2 cm, DSS_Rabex^LD^ resulted in a colon length of 6.3 cm, and no significant difference was observed compared to the non-treated normal group. This indicates that SSZ and Rabex protected the colon and inhibited colon shrinkage caused by DSS (Figure 2D-E). Moreover, the degree of DAI score increase after DSS treatment was reduced to 3.5 in the DSS_SSZ, and to 5.2 in the DSS_Rabex^LD^ (Figure 2F), compared to 7.8 in the DSS_CTRL group on day 14 after DSS treatment (Figure 2G). Histology of mouse colons was performed by H&E staining to compare the three groups. Compared to the normal CTRL group, colitis induced by DSS resulted in specific characteristics within the colon tissue of the DSS_CTRL group, including increased cellular infiltration, goblet cell depletion, and greater distortion/damage to the crypt architecture (Figure 2H). However, in the group undergoing oral SSZ and Rabex^LD^ treatment, minimal levels of cellular infiltration and reduced loss of colon tissue were noted compared to those in the DSS_CTRL group. Overall, Rabex exhibited therapeutic activity against IBD, and oral administration of Rabex effectively restored damaged colons.

To assess the stability of Rabex after oral administration, an *in vitro* digestion test was performed (Figure S5A). In the *in vitro* digestion test, the pH was adjusted, and enzymes and bile salts were added to simulate gastrointestinal (GI) conditions 50, 51. Rabex was first incubated in a stomach-like solution at 37°C, and incubation was then continued in an intestinal-like solution. The Rabex concentration decreased by 20.1% when the Rabex were incubated in a stomach-like solution (Figure S5B). After reaction with the intestinal solution, the final Rabex concentration was 38.6%. Additionally, the size of Rabex increased from an initial average size of 119.8 nm before incubation to 158.5 nm in the stomach-like solution and 155.7 nm in the intestinal-like solution (Figure S5C). The results revealed that a large amount of Rabex remained intact even after exposure to the two subsequent environments.

### Rabex regulation of macrophage inflammation and its promotion of colon epithelial cell regeneration

Immune cells assemble within the colons of patients with IBD and secrete inflammatory cytokines, thus initiating an inflammatory response 1. When intestinal inflammation occurs, it is essential to simultaneously suppress excessive immune cell responses and maintain healthy colonic epithelial cells 6, 52. Thus, therapeutic efficacy can be maximized if a drug candidate for IBD possesses dual functions that simultaneously inhibit inflammation in macrophages and promote colon epithelial cell regeneration. As we provided evidence that Rabex effectively cured the colons of a mouse *in vivo* IBD model, we performed *in vitro* analysis to determine if Rabex possesses the dual functionality required for effective IBD treatment.

**Figure 3:**
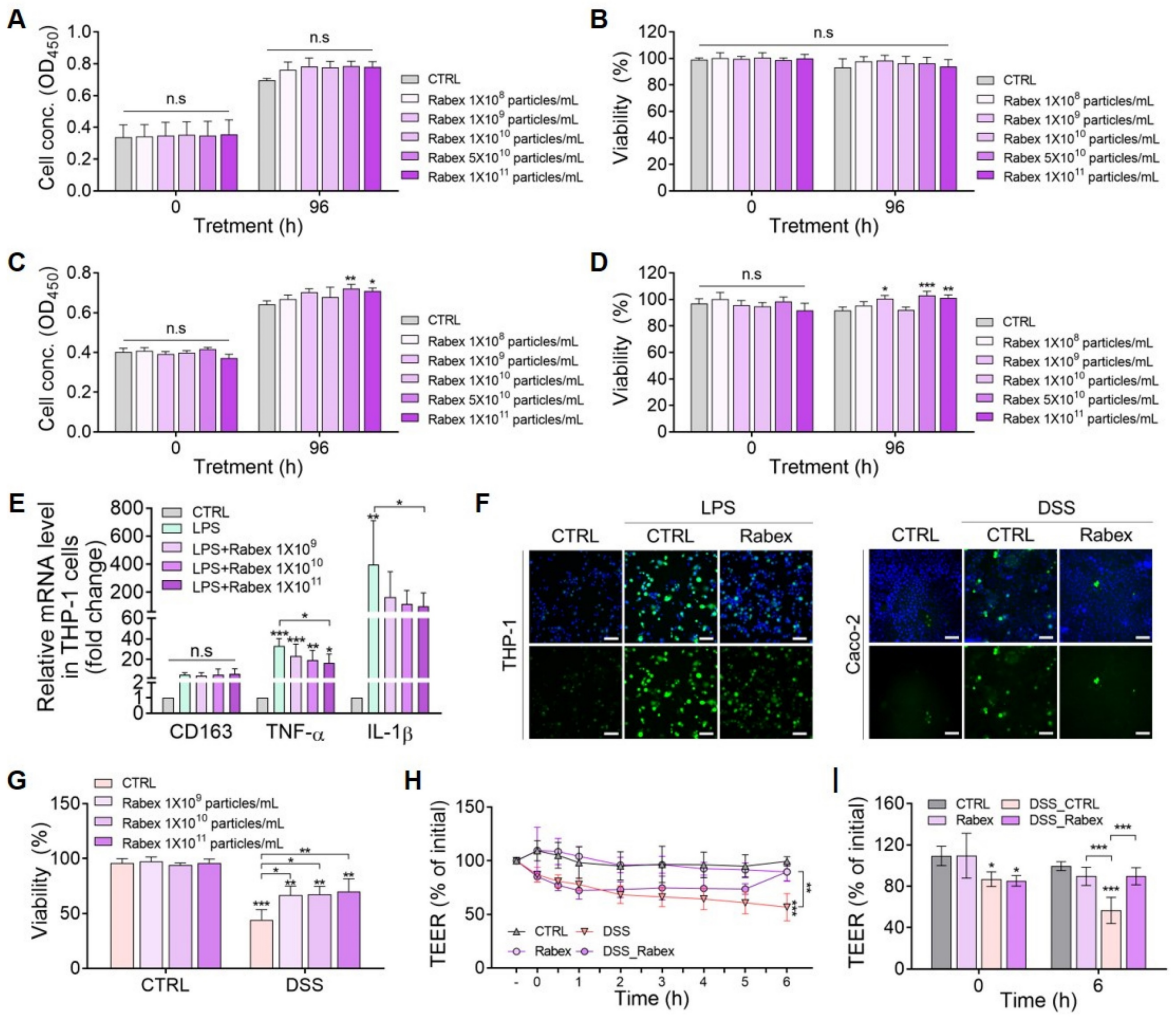
** Regulation of macrophage inflammation and promotion of epithelial cell regeneration by Rabex.** (**A**, **B**) Effect of Rabex on Caco-2 colon epithelial cell proliferation (**A**) and cytotoxicity (**B**). (**C**, **D**) Effect of Rabex on THP-1 macrophage proliferation (**C**) and cytotoxicity (**D**). Different concentrations of Rabex were administered to each cell line, and Rabex promoted the proliferation of both cell lines without exhibiting toxicity. (**E**) Dose-dependent inhibition of inflammation by Rabex in THP-1 cells treated with LPS. Cells were treated the LPS for the induction of inflammation in the absence or presence of Rabex. Anti-inflammatory effects were assessed using RT-PCR analysis of M1 polarization markers. (**F**) Antioxidative effect of Rabex was assessed in THP-1 and Caco-2 cells treated with LPS and DSS, respectively, for ROS generation. ROS level was detected by DCF-DA staining, with size bars indicating 100 μm. (**G**) Inhibition of DSS-induced cell death in Caco-2 cells by Rabex. (**H**, **I**) Effect of Rabex on DSS-induced paracellular permeability as demonstrated by the impact of Rabex on the TEER of a Caco-2 cell monolayer. Rabex treatment was observed to prevent changes in TEER induced by DSS. The data are presented as mean ± SEM, n ≥ 3. Statistical significance is indicated as follows: *p < 0.05; **p < 0.01; ***p < 0.001; n.s, not significant.

First, the dose-dependent effects of Rabex on cell proliferation and cytotoxicity in Caco-2 colon epithelial cells and THP-1 macrophages were evaluated (Figure 3A-D). Various doses of Rabex (1 × 10^8^ to 1 × 10^11^ particles/mL) were added to the cell culture medium, and cell proliferation was observed in both cell types, particularly at high doses of Rabex (Figure 3A, C). Moreover, no cytotoxicity was observed in either cell line, even when high doses of Rabex were administered for 96 h (Figure 3B, D). These results demonstrated that Rabex promotes the proliferation of both Caco-2 and THP-1 cells without exhibiting toxicity.

We further investigated the biological functions of Rabex in the regulation of inflammation in macrophages. Monocyte THP-1 cells were differentiated into macrophages (M0) using phorbol 12-Myristate 13-Acetate (PMA), and this was followed by lipopolysaccharide (LPS) treatment for 24 h to induce inflammation after supplementation with different doses of Rabex ranging from 1 × 10^9^ to 1 × 10^11^ particles/mL. qRT-PCR results revealed that the expression of the M0 marker CD163 in both LPS-treated and the groups treated with Rabex followed by LPS exhibited no significant difference compared to that of the untreated CTRL cells (Figure 3E). When macrophages were treated with LPS in the absence of Rabex, the expression of M1 markers, including TNF-α and IL-1β (pro-inflammatory factors), drastically increased by 33.1- and 395-fold, respectively. However, the increase in these pro-inflammatory factors was significantly reduced when the cells were supplemented with Rabex. Dose-dependent decreases were observed, and TNF-α and IL-1β increased in the group treated with 1 × 10^11^ particles/mL Rabex followed by LPS treatment by only 16.6- and 99-fold. This corresponded to 49.8 and 74.9% reductions, respectively. These findings suggest that Rabex effectively suppresses LPS-induced pro-inflammatory gene expression in macrophages.

As reactive oxygen species (ROS) generation in cells under IBD environments severely damages the cells, the anti-oxidative effects of Rabex in THP-1 and Caco-2 cells were tested (Figure 3F) 53, 54. Induction of inflammation in THP-1 cells resulted in increased ROS production, as observed by fluorescent microscopy after DCF-DA staining. A substantial decrease in ROS generation was observed in cells supplemented with Rabex that possesses the intrinsic ability to scavenge ROS. Colonic epithelial cells also generate ROS in response to inflammation. Similar to THP-1 cells, reduced ROS levels were detected in cells treated with Rabex disulfide DSS.

To assess the ability of Rabex to protect colon epithelial cells, we induced cell death in Caco-2 cells using DSS. Cells were pretreated with 5% DSS for 24 h and then cultured for 96 h in the presence of different concentrations of Rabex (1 × 10^9^ to 1 × 10^11^ particles/mL). The WST-1 assay revealed that over 66.5% viability was observed in cells treated with Rabex, whereas only 44.2% of cells without Rabex survived (Figure 3G).

Disruption of tight junctions in the intestinal epithelium in patients with IBD increases permeability and promotes immune cell infiltration, thereby enhancing inflammation in the colon 55, 56. Thus, transepithelial electrical resistance (TEER) was monitored to assess the effects of Rabex on the DSS-induced intestinal epithelial barrier (Figure 3H-I). TEER values gradually decreased with DSS treatment, reaching 56.7% at 6 h after treatment. In contrast, Rabex supplementation maintained tight junctions with a TEER value of 89.7%, and no significant difference was observed compared to that of untreated normal cells. This result indicated that Rabex was capable of restoring tight junctions by preserving TEER values. Overall, Rabex inhibited inflammatory gene expression and ROS generation in immune cells, protected colon epithelial cells from death, and maintained tight junctions within the intestinal epithelium. Thus, Rabex is a promising drug candidate with the dual functionality required for effective IBD treatment.

### Gene expression profiling in colon tissue from DSS-induced mouse colitis model

**Figure 4:**
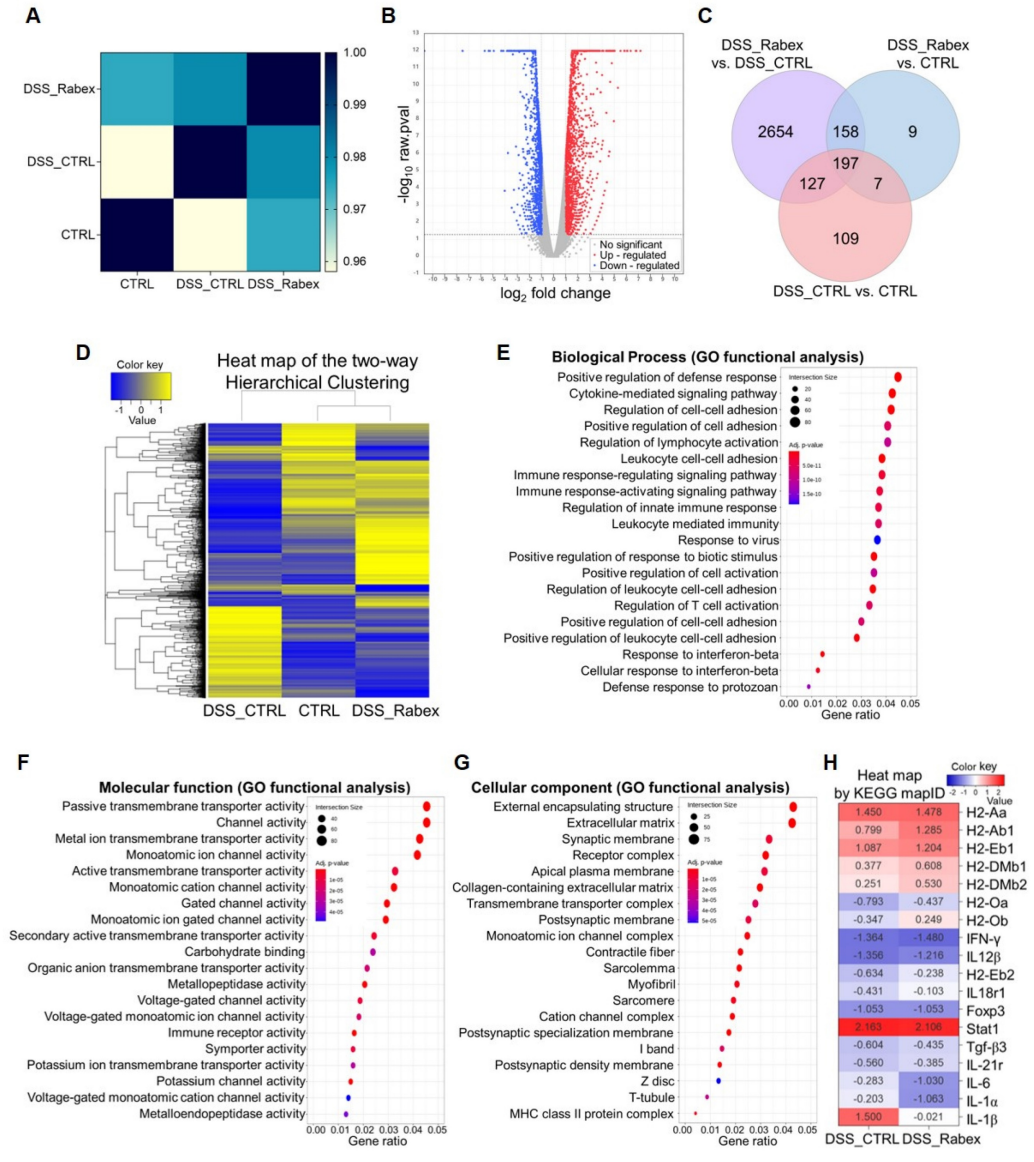
** mRNA expression profiling in colon tissues of DSS-induced mouse colitis models.** (**A**) Pearson's correlation coefficient analysis indicating the degree of similarity among CTRL, DSS_CTRL, and DSS_Rabex groups. Note that high similarity was observed between CTRL and DSS_Rabex groups. (**B**) Volcano plot comparing gene expressions in DSS_Rabex and DSS_CTRL groups. The red and blue dots indicate up-regulated and down-regulated genes. (**C**) Venn diagram illustrating gene expression correlations. (**D**) Hierarchical clustering analysis of differentially expressed genes in CTRL, DSS_CTRL, and DSS_Rabex groups. Note that Rabex administration to DSS-induced mouse exhibited a similar expression pattern compared to that of the normal CTRL group. (**E**-**G**) Gene ontology analysis identifying genes related to biological process (**E**), molecular function (**F**), and cellular component. (**G**) in DSS_Rabex versus DSS_CTRL (**H**) KEGG pathway analysis revealing differential gene expression in the IBD pathway between DSS_CTRL and DSS_Rabex groups.

To understand the effects of Rabex in the DSS-induced colitis model, mRNA sequencing analysis of colon tissues isolated from mice treated with or without Rabex was performed. Using a p-value cutoff of < 0.05 and normalized values for each sample, we examined the degree of similarity (Pearson's coefficient) between samples to verify the reproducibility of replicate samples (Range: -1 ≤ r ≤ 1). A correlation coefficient closer to 1 indicates a higher similarity between samples. A high correlation (0.97 between the CTRL (normal) and DSS_Rabex groups) was observed, and this was higher than that between the CTRL and DSS_CTRL groups (Figure 4A). RNA sequencing analysis revealed 1,807 upregulated and 1,329 downregulated genes in the volcano plot comparing the DSS_CTRL and DSS_Rabex groups (Figure 4B). The overall correlations between groups are illustrated using a Venn diagram (Figure 4C) and hierarchical clustering (Figure 4D). Hierarchical clustering analysis (Euclidean distance, complete linkage) was employed to group and analyze samples and genes with similar expression levels using normalized values for each gene that were significant across at least one comparison group. Upon analyzing 3,261 genes, a high degree of similarity was observed between the CTRL and DSS_Rabex groups, whereas the CTRL and DSS_CTRL groups exhibited lower similarity.

To verify the functionality of Rabex, we conducted comprehensive analysis using Gene Ontology (GO) and KEGG pathway analyses. GO analysis targeted genes with significant differences in expression levels and examined three categories that included biological processes (Figure 4E), molecular functions (Figure 4F), and cellular components (Figure 4G). Compared to the DSS_CTRL group, the DSS_Rabex group exhibited a significant presence of genes associated with the positive regulation of the defense response (e.g. INF-γ), extracellular matrix (e.g. IGFBP6, Myoc), and collagen-containing extracellular matrix (e.g. Col19a1, Col22a1). Concurrently, KEGG pathway analysis clearly indicated that the expression levels of genes involved in the IBD pathway, including IL-6, IL-1α, and IL-1β, were notably lower in DSS_Rabex than they were in DSS_CTRL (Figure 4H). Thus, Rabex delivered to the colon regulates the expression of genes related to colitis.

### Surface modification of Rabex with hyaluronic acid for enhanced targeted therapy

**Figure 5:**
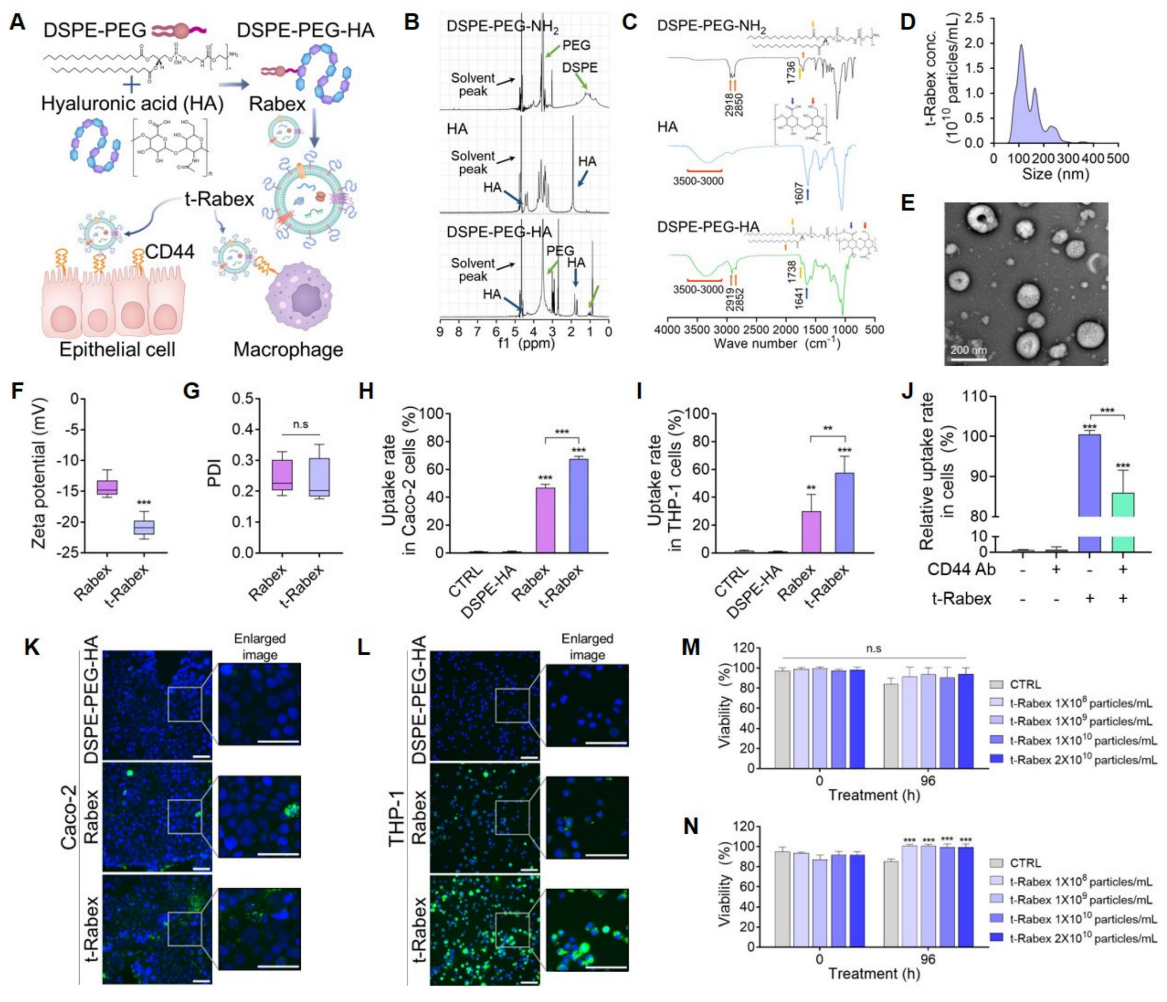
** Surface engineering of Rabex for enhanced targeted therapy.** (**A**) Schematic illustration of DSPE-PEG-HA conjugate to Rabex to construct t-Rabex. (**B**) ^1^H-NMR spectroscopy confirming DSPE-PEG-HA synthesis and highlighting HA and DSPE-PEG-NH_2_ peaks. (**C**) FT-IR spectra of HA, DSPE, and DSPE-PEG-HA conjugate. Orange arrows: methylene groups of DSPE; Yellow arrows: carbonyl groups of DSPE; Red arrows: hydroxyl groups of HA and amine groups of DSPE; Blue arrows: carboxylic groups of HA. (**D**-**G**) Characterization of t-Rabex for their size (**D**), morphology (**E**), Zeta potential (**F**), and PDI (**G**). Zeta potential and PDI were compared between Rabex and t-Rabex. (**H**) Flow cytometric analysis demonstrating t-Rabex delivery to Caco-2 cells. Rabex and t-Rabex were stained with PKH staining dye, and DSPE-PEG-HA without Rabex was used as negative control. Note that a higher number of t-Rabex was uptaken by colon epithelial cells compared to that of Rabex. (**I**) Flow cytometric analysis of t-Rabex in THP-1 cells indicating surface engineered t-Rabex exhibited enhanced cellular uptake in both cells. (**J**) The CD44 receptor targeting efficiency of t-Rabex was assessed using flow cytometry on THP-1 cells, both with and without the CD44 blocking antibody. (**K**, **L**) Fluorescent microscopy demonstrating the enhanced t-Rabex uptake into Caco-2 (**K**) and THP-1 cells (**L**), with size bars indicating 100 μm. Free DSPE-PEG-HA without Rabex (upper panel), Rabex (middle panel), and t-Rabex (lower panel) were imaged for the comparison. (**M**, **N**) Assessment of cytotoxicity of t-Rabex using the WST-1 assay. Note that t-Rabex exhibited no cytotoxicity in Caco-2 (**M**) and THP-1 (**N**) cells. The data are presented as mean ± SEM, n ≥ 3. Statistical significance is indicated as follows: **p < 0.01; ***p < 0.001; n.s, not significant.

One of the major challenges in current IBD treatment strategies is the delivery of therapeutic agents to the inflamed regions of the colon. However, in IBD, factors such as the gastrointestinal pH, transit time, and alterations in the microbiome make drug release unpredictable and lead to unsatisfactory therapeutic efficacy. Therefore, targeted drug delivery to the inflamed intestinal areas requires strategic approaches. Moreover, individual variations in the onset severity highlight the need to develop potent medications capable of addressing the diversity of this condition. To enhance the therapeutic effects, engineering efforts have been undertaken to enable the targeting effects of Rabex on immune and intestinal epithelial cells when orally administered. Specifically, the surfaces of the immune and intestinal epithelial cells are rich in CD44 receptors during colitis 57, 58. In particular, immune cells such as macrophages are known to express higher levels of CD44 when polarized as M1 cells 59. CD44 is a receptor that is responsible for lymphocyte activation, recirculation, homing, and hematopoiesis. As hyaluronic acid (HA) can be effectively utilized as a ligand for CD44 in IBD therapy, Rabex was engineered to display HA on its surface (Figure 5A) 60-63.

To conjugate HA onto the Rabex surface, the carboxylic group of HA was first coupled with the amine group of 1,2-Distearoyl-sn-glycero-3-phosphoethanolamine (DSPE)-polyethylene glycol (PEG)-NH_2_ that could bind to the lipid membrane of EVs by the insertion of DSPE. The detailed conjugation process is described in the Materials & Methods. After removing unreacted impurities using dialysis, nuclear magnetic resonance spectroscopy (^1^H-NMR) confirmed the presence of both the-NHCOCH_3_ group of HA and the-CH_2_CH_2_O group of DSPE-PEG-NH_2_ in DSPE-PEG-HA (Figure 5B). The Fourier-transform infrared (FT-IR) spectra of DSPE-PEG-NH_2_, HA, and DSPE-PEG-HA confirmed successful formation of the DSPE-PEG-HA conjugate (Figure 5C). To rule out the possibility of DSPE-PEG-HA spontaneously forming micelles in solution rather than binding to the lipid membrane of Rabex, the optimal concentration of DSPE-PEG-HA for binding to Rabex without micelle formation was assessed 64.

Various concentration of DSPE-PEG-HA (0.01 to 0.5 mg/mL) in the absence of Rabex were incubated, and negligible levels of particles were observed (particularly for 0.01 or 0.05 mg/mL, Figure S6A). Particle formation was observed using NTA and TEM, confirming that no particles were formed at a concentration of 0.01 mg/mL DSPE-PEG-HA (Figure S6B-C). Thus, 0.01 mg/mL was chosen for surface modification of Rabex. To insert DSPE-PEG-HA onto the surface of Rabex, the reaction was performed at 37°C for 4 hours, and this was followed by separation of unbound DSPE-PEG-HA using ultrafiltration. The resulting product consisting of DSPE-PEG-HA bound to Rabex was named t-Rabex. The binding of HA to Rabex via lipid membrane insertion was demonstrated by incubating DSPE-PEG-Cy5 with Rabex stained with PKH dye. Fluorescence microscopy revealed co-localization of PKH and Cy5 signals, thus indicating that t-Rabex was successfully constructed (Figure S7A-C). To further substantiate that DSPE-PEG-HA coupling to the surface of Rabex, FT-IR spectra of DSPE-PEG-HA, Rabex, and t-Rabex was performed (Figure S8). The analysis revealed a distinct peak at 1,738 cm^-1^ in t-Rabex, which was absent in Rabex but present in DSPE-PEG-HA. This peak confirms the coupling of DSPE-PEG-HA to Rabex. NTA and TEM results revealed that the size of t-Rabex was 142.5 nm, and HA conjugation did not change the size or shape of Rabex (Figure 5D-E). The zeta potential of t-Rabex was altered from -14.4 ± 1.48 mV to -20.9 ± 1.41 mV due to the surface binding of HA (Figure 5F), while the PDI value remained consistent before and after engineering, thus confirming a uniform dispersion (Figure 5G). To assess the stability of t-Rabex after oral administration, an *in vitro* digestion test was performed, similar to the method used in Figure S5 (Figure S9A). The t-Rabex concentration decreased by 23.7% when incubated in a stomach-like solution (Figure S9B). After reacting with the intestinal solution, the final t-Rabex concentration decreased by 27.0%. Additionally, the size of t-Rabex increased from an initial average of 119.6 nm before incubation to 146.4 nm in both the stomach-like and intestinal-like solutions (Figure S9C). These results demonstrate that, like Rabex, t-Rabex can also be delivered to the colon after passing through the GI tract following oral administration.

### Assessment of t-Rabex targeting ability toward colon epithelial cells and macrophages

The enhanced targeting of t-Rabex was directly confirmed in Caco-2 and THP-1 cells by flow cytometry and fluorescence microscopy. First, PKH-labeled DSPE-PEG-HA, Rabex, and t-Rabex were prepared and incubated with Caco-2 cells at the same concentration (1 × 10^10^ particles/mL) for 24 h. The cellular uptake of each nanoparticle was analyzed by flow cytometry (Figure S10A). A large shift in fluorescence intensity was observed for cells incubated with Rabex compared to that of the control and DSPE-HA-treated cells, thus indicating that a high amount of Rabex was taken up by Caco-2 cells. The fluorescence intensity shifted further when the cells were incubated with t-Rabex, thus indicating that HA conjugation on the surface of Rabex enhanced its cellular uptake. The delivery efficiency was calculated as a percentage relative to the control. The Rabex delivery efficiency to Caco-2 cells was 46.9%, while that of t-Rabex was 67.6% (Figure 5H). Similar results were obtained when THP-1 macrophages were incubated with t-Rabex (Figure S10B). The delivery efficiency of Rabex was 29.9%, whereas that of t-Rabex was 57.8% (Figure 5I). These results revealed that surface-engineered t-Rabex demonstrated a higher degree of delivery through the CD44 targeting of HA. Additionally, it is noteworthy that macrophages exhibited a greater increase in targeting efficiency than that of intestinal epithelial cells, due to the observation that they exhibit higher CD44 expression than do intestinal epithelial cells 59. To confirm that the targeting effect of t-Rabex is mediated by the binding of HA to the CD44 receptor, the CD44 receptor was blocked with a CD44 antibody, and the targeting efficiency of t-Rabex was analyzed using flow cytometry (Figure 5J). The relative uptake rate of fluorescence labeled t-Rabex in CD44 antibody untreated THP-1 cells was 100.5%, which decreased to 86.0% when the CD44 receptor was blocked. This demonstrates that HA conjugation to Rabex enhanced the targeting efficiency to the CD44 receptor. To further verify the enhanced cell-targeting efficiency of t-Rabex, Caco-2 and THP-1 cells were treated with fluorescently labeled t-Rabex. As presented in Figure 5K and L, a significantly higher uptake of t-Rabex was observed in both cell lines than that for unengineered Rabex. This was particularly evident in THP-1 cells, where although Rabex demonstrated decent delivery, incubation with t-Rabex resulted in almost all cells exhibiting green fluorescence.

To assess if t-Rabex exhibited cytotoxicity, we incubated Caco-2 and THP-1 cells with varying concentrations of t-Rabex and analyzed their viability using the WST-1 assay. In Caco-2 cells, t-Rabex maintained high viability similar to that of the non-treated control group, thus demonstrating that t-Rabex does not exhibit toxicity (Figure 5M). We observed that the group treated with t-Rabex at a concentration of 1 × 10^8^ to 2 × 10^10^ particles/mL exhibited 13.6% higher viability than that of the control group (Figure 5N). These results indicated that the engineered t-Rabex was not cytotoxic and supported the proliferation and survival of THP-1 cells.

### Validating the enhanced in vivo therapeutic efficacy of t-Rabex in an IBD model

As demonstrated previously, oral administration of Rabex successfully recovered the damaged colon and treated colitis in an IBD model (Figure 2). Subsequently, *in vitro* studies have demonstrated that Rabex retains its dual functionality, thus enabling the protection of intestinal epithelial cells and the inhibition of macrophages (Figure 3). Rabex was conjugated to HA to generate t-Rabex, and this enhanced the targeting of intestinal epithelial cells and macrophages (Figure 5). Herein, we evaluated the therapeutic efficacy of t-Rabex compared to that of nonengineered Rabex to demonstrate its enhanced efficacy in IBD treatment. To demonstrate the improved therapeutic efficacy of t-Rabex, 5 × 10^10^ particles/mL of t-Rabex (referred to as t-Rabex^LD^) were administered, whereas 5 × 10^11^ particles/mL of Rabex (referred to as Rabex^HD^) that is 10-fold higher was used for comparison.

**Figure 6:**
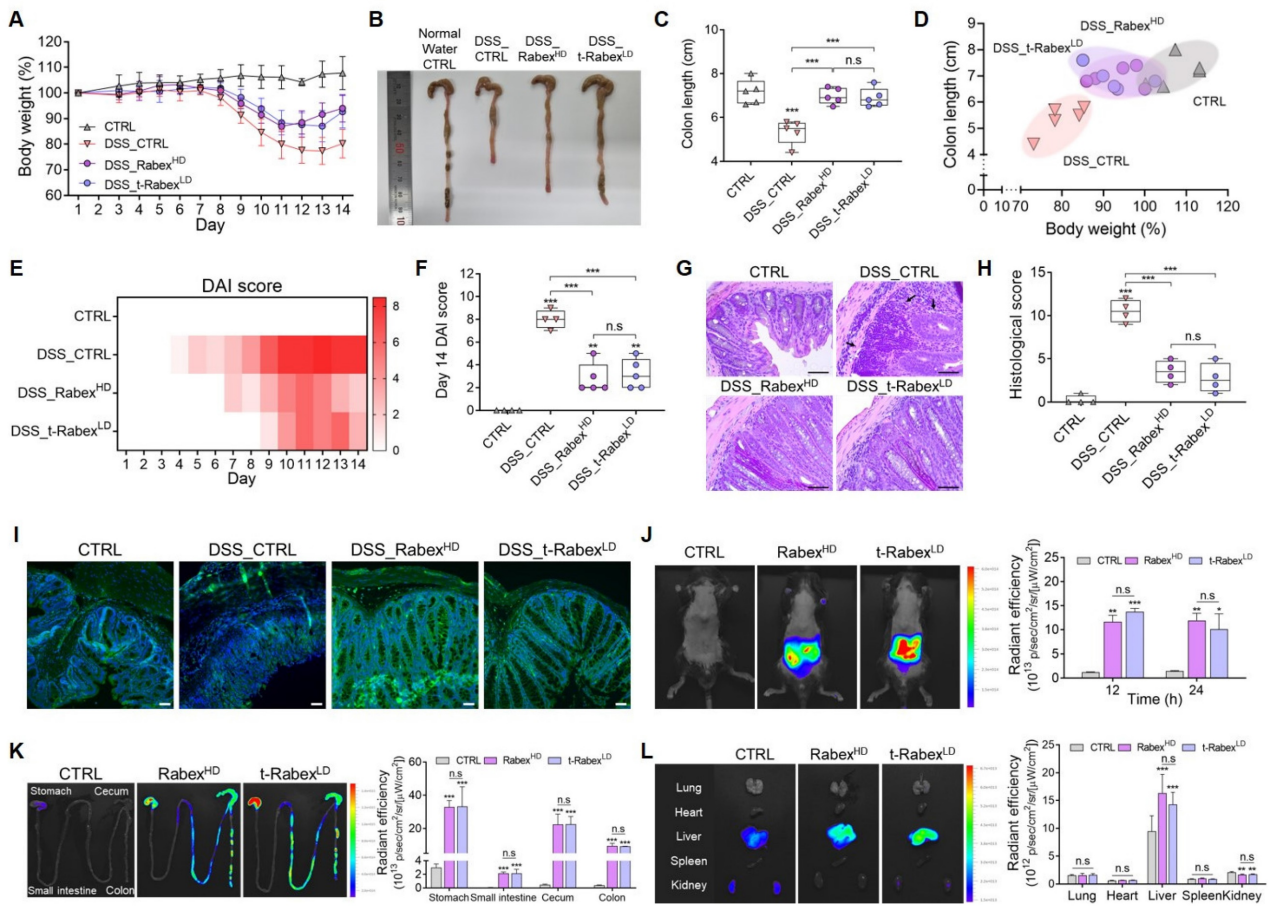
***In vivo* therapeutic efficacy and biodistribution of t-Rabex in an IBD model.** (**A**) The comparison of body weights among the CTRL, DSS_CTRL, Rabex^HD^, and t-Rabex^LD^ groups in DSS-treated mice. Note that 5 × 10^10^ particles/mL of t-Rabex (t-Rabex^LD^) were used for assessment, whereas 5 × 10^11^ particles/mL of Rabex (Rabex^HD^) (10-fold higher) were used for the comparison. (**B**, **C**) Effect of t-Rabex administration on the colon length. (**D**) Graph indicating correlation between body weight loss and colon length in the colitis model. Note that both Rabex^HD^ and t-Rabex^LD^ resulted in the population clearly resembling that of the normal CTRL group (**E**, **F**). Heat map indicating DAI scores on day 14 for each group. (**G**) H&E staining of colon tissues indicating cell distribution in inflamed regions (black arrow), with size bars indicating 50 μm. (**H**) Histological scoring comparison among CTRL, DSS_CTRL, DSS_Rabex^HD^, and DSS_t-Rabex^LD^ groups. (**I**) Immunohistochemistry of ZO-1 in colon tissues demonstrating tight junction integrity, with size bars indicating 100 μm. (**J**-**K**) Biodistribution of Rabex and t-Rabex. Both EVs were stained with DiD membrane staining dye and orally administrated for 24 h. (**J**) Fluorescence imaging of the ventral area (Left images) and quantitative analysis indicating radiant efficiency of Rabex^HD^ and t-Rabex^LD^ (Right graph). (**K**) *In vivo* tracing of of Rabex^HD^ and t-Rabex^LD^ across the GI tract and quantitative analysis indicating radiant efficiency for each sections (stomach, small intestine, cecum, and colon). (**L**) Fluorescent imaging and the resulting radiant efficiency in major organs (lungs, heart, liver, spleen, and kidneys). The data are presented as mean ± SEM, n ≥ 3. Statistical significance is indicated as follows: *p < 0.05; **p < 0.01; ***p < 0.001; n.s, not significant.

To evaluate the therapeutic efficacy of t-Rabex^LD^ in an *in vivo* colitis model, administration protocols for DSS and Rabex were identical to those for Rabex^LD^ (Figure 2A). The body weight of DSS-treated mice (DSS_CTRL group) decreased by 19.8% on day 14 (Figure 6A, Figure S11A). However, the groups that were orally supplemented with Rabex- (DSS_Rabex^HD^) and t-Rabex-treated groups treated with DSS (DSS_t-Rabex^LD^) displayed significantly milder weight reductions of 6.0% and 7.3%, respectively. Colon length analysis revealed that the DSS_CTRL group treated with normal water exhibited a reduction from 7.2 cm to 5.3 cm after DSS administration (Figure 6B-C, Figure S11B). In contrast, the DSS_Rabex^HD^ and DSS_t-Rabex^LD^ groups exhibited less pronounced reductions, with colon lengths of 7.0 cm and 6.9 cm, respectively. Notably, considering that a lower EV dose was used for t-Rabex^LD^ (5 × 10^10^ particles/mL) than for Rabex^HD^ (5 × 10^11^ particles/mL), Rabex modified with a targeting function enabled enhanced therapeutic treatment of IBD. The correlation between body weight loss and colon length reduction in the colitis model exhibited a concurrent decrease in colon length and body weight with the acceleration of IBD due to DSS treatment (Figure 6D). However, when Rabex^HD^ and t-Rabex^LD^ were administered orally, the population clearly resembled that of the CTRL group, thus indicating the mitigation of these major outcomes in IBD. DAI scores on day 14 were 8 in the DSS_CTRL group, while the DSS_Rabex^HD^ and DSS_t-Rabex^LD^ groups displayed lower scores of 2.9 and 3.2, respectively (Figure 6E-F).

H&E staining of mouse colon tissues was performed to investigate the effect of t-Rabex on cell distribution in the inflamed region of the colon (Figure 6G). Compared to the CTRL group, DSS treatment induced subcutaneous immune cell infiltration and colonic epithelial tissue loss. Upon measuring the histological scores, we observed that while the normal CTRL group possessed a score of 0.25, the score escalated to 10.5 in the DSS_CTRL group upon DSS treatment (Figure 6H). In contrast, both the Rabex- and t-Rabex-treated groups treated with DSS exhibited histological staining results similar to those of the normal CTRL group. In the DSS_Rabex^HD^ group, the histological score decreased to 3.5, and in the DSS_t-Rabex^LD^ group, it was further reduced to 2.75. These results demonstrate that t-Rabex^LD^ effectively suppresses the progression of IBD.

To assess the therapeutic effect of t-Rabex at a high dose, t-Rabex^HD^ was administered orally for 10 doses at a concentration of 5 × 10^11^ particles/mL, identical to Rabex^HD^. As shown in Figure S11C-E, the recovery rate of body weight was faster compared to other groups, with a DAI score of 1.2. The colon length was 6.7 cm (Figure S11F), and H&E staining of colon tissue showed a structure similar to that of normal tissue (Figure S11G). These findings demonstrate the enhanced therapeutic efficacy of t-Rabex^HD^. However, considering the amount of Rabex required to engineer t-Rabex^HD^, t-Rabex^LD^ is sufficient for effective IBD treatment.

To evaluate the efficacy of t-Rabex in a setting where patients have already been diagnosed with IBD, after administering DSS for 5 days, Rabex^HD^ and t-Rabex^LD^ were orally administered in 10 doses to evaluate their effects (Figure S12A). As a result, on day 16, both the DSS_Rabex^HD^ and DSS_t-Rabex^LD^ groups had lower DAI scores and showed a faster recovery rate in body weight compared to the DSS_CTRL group (Figure S12B-C). Additionally, the colon length in the DSS_t-Rabex^LD^ group recovered to 6.5 cm, compared to 5.1 cm in the DSS_CTRL group (Figure S12D-E), H&E staining of mouse colon tissues also showed a higher degree of tissue recovery in the DSS_Rabex^HD^ and DSS_t-Rabex^LD^ groups (Figure S12F). These results confirm that Rabex can demonstrate therapeutic efficacy both before and after the onset of IBD induced by DSS.

Figure 6I presents the immunohistochemistry of ZO-1, a molecule involved in tight junctions, in the colon. Compared to the CTRL group, ZO-1 levels drastically decreased after DSS treatment in the DSS_CTRL group as expected. In contrast, both the DSS_Rabex^HD^ and DSS_t-Rabex^LD^ groups exhibited fluorescent intensities of ZO-1 comparable to those of the normal CTRL group. These results confirmed that t-Rabex^LD^ at a dosage ten times lower than that of Rabex^HD^ exerts a similar therapeutic effect for treating colitis. Thus, Rabex engineered for enhanced targeting promotes the delivery of active gradients for IBD treatment.

### Biodistribution study of Rabex and t-Rabex following oral administration in mice

To assess the biodistribution of Rabex and t-Rabex after oral administration, fluorescence imaging was performed in the ventral area, colon, and other organs of the mice using an IVIS (*In Vivo* Imaging System) 65, 66. Both Rabex^HD^ and t-Rabex^LD^ were labeled with a DiD membrane-staining dye for fluorescence imaging. Rabex^HD^ and t-Rabex^LD^ were orally administered to mice. A higher fluorescence value in the ventral area was observed for both EVs, and the radiant efficiencies for Rabex^HD^ and t-Rabex^LD^ were 11.9 and 12.2, respectively, after 24 h after administration (Figure 6J). The fluorescence values were also compared across different sections of the gastrointestinal (GI) tract, including the stomach, small intestine, cecum, and colon (Figure 6K). Forty-eight hours post-administration, the fluorescence values in the ventral area (Figure S13A-B) and colon (Figure S13C-D) were observed to decrease. This indicates that in all sections, both Rabex^HD^ and t-Rabex^LD^ exhibited higher fluorescence than did CTRL, confirming their absorption. Thus, indicating their effective distribution throughout the GI tract. Additionally, no significant difference between Rabex^HD^ and t-Rabex^LD^ was observed for the biodistribution in the GI tract. Considering that a 10-fold lower dose of t-Rabex was used, t-Rabex exhibited a higher delivery efficiency than did Rabex, and this in turn revealed enhanced therapeutic efficacy for IBD treatment. Upon analyzing the radiant efficiency in the lungs, heart, liver, spleen, and kidneys, it was observed that the liver exhibited the highest fluorescence values for Rabex^HD^ and t-Rabex^LD^ at 16.3 and 14.3, respectively (Figure 6L, Figure S13E-F). These results suggested that orally administered Rabex^HD^ and t-Rabex^LD^ were effectively transported along the GI tract and delivered to the liver via the portal vein. These results confirm that orally administered Rabex was effectively delivered to the intestine. Additionally, t-Rabex that was modified to target CD44 in intestinal epithelial and immune cells demonstrated an enhanced targeting effect in areas of the intestine. This highlights the potential of t-Rabex for precise drug delivery to inflamed regions of the GI tract.

### Understanding the mechanism of colitis suppression through profiling gene expression in the colons and the biocompatibility of t-Rabex

To gain insights into the mechanisms underlying the therapeutic effects of Rabex in DSS-induced colitis, we performed qRT-PCR analysis of colonic tissues. Based on the previously reported colitis study that DSS treatment resulted in significantly higher levels of inflammatory markers in the distal colon compared to the proximal colon, the colon located closer to the cecum was specifically designated as the “proximal colonˮ, whereas the region closer to the anus was referred to as the “distal colonˮ (Figure S11B) 67-70. By dividing the colon into these two sections and performing separate RNA analyses, the mechanism by which t-Rabex suppresses colitis was elucidated. RNA was extracted from both the colonic tissue specimens. We systematically classified the pivotal cellular responses at the onset of colitis into three primary categories that included anti-inflammatory, anti-oxidant, and tight junction maintenance 71, 72. To evaluate the anti-inflammatory function, pro-inflammatory markers associated with M1 polarization, including IL-1β, IL-6, TNF-α, and COX-2, and anti-inflammatory markers associated with M2 polarization, including CD206 and Arg-1, were selected. To assess anti-oxidative properties, NQO-1, Nrf-2, and HO-1 were assessed, while ZO-1, Claudin-1, and Occludin were chosen for the evaluation of tight junction maintenance.

**Figure 7:**
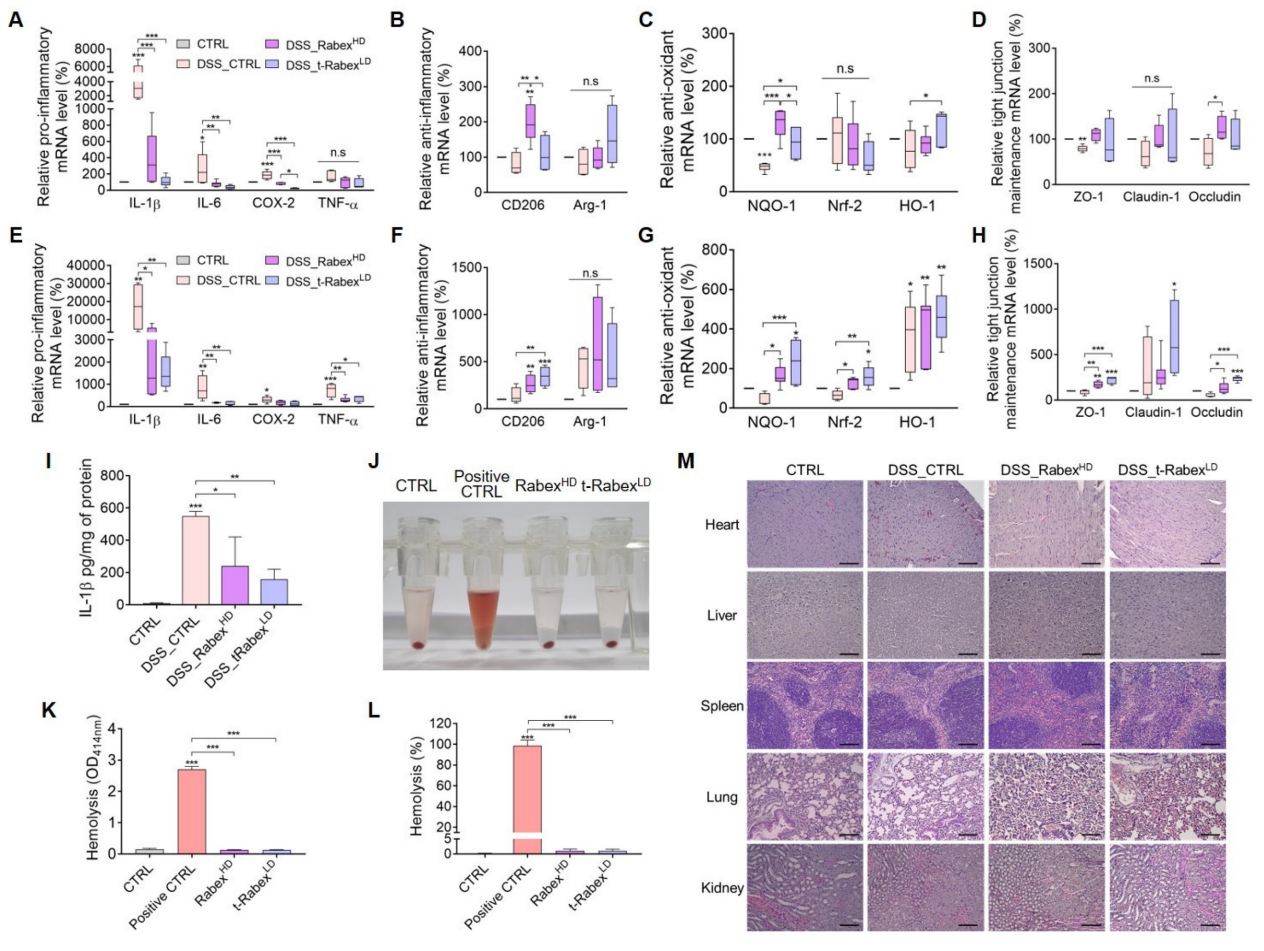
** RNA expression profiling and biocompatibility assessment of Rabex and t-Rabex in proximal and distal colons of DSS-induced colitis models.** RNAs were extracted from both sections for RT-PCR analysis. RT-PCR analysis of gene expression in the proximal colon. (**A**,** E**) RT-PCR analysis of gene expression in the proximal and distal colon, including pro-inflammatory genes (IL-1β, IL-6, Cox-2, and TNF-α), for CTRL, DSS_CTRL, DSS_Rabex^HD^, and DSS_t-Rabex^LD^ groups in each section. (**B**, **F**) Anti-inflammatory genes (CD206 and Arg-1) were analyzed for each group in the proximal and distal colon, respectively. (**C**,**G**) Anti-oxidant effects of Rabex and t-Rabex were assessed using RT-PCR of NQO-1, Nrf-2, and HO-1 in the proximal and distal colon, respectively. (**D**, **H**) Tight junction maintenance-related genes (ZO-1, Claudin-1, and Occludin) were analyzed in the proximal and distal colon, respectively. (**I**) ELISA analysis of IL-1β protein levels in the entire colon tissue. (**J**) Representative photograph of the sample post-hemolysis experiment, following centrifugation for the hemolysis measured. The pellet in the bottom of tube indicate the precipitated RBCs after centrifugation. (**K**) Determination of hemolysis by measuring the absorbance at 414 nm of each supernatant after centrifugation. (**L**) Determination of hemolysis percentage.양식의 맨 위 (**M**) Histological analysis of various organs (heart, liver, spleen, lungs, and kidneys) on day 14 for evaluating the biocompatibility of Rabex^LD^, Rabex^HD^, and t-Rabex^LD^ with results derived from H&E staining. Size bars indicate 100 μm. The data are presented as mean ± SEM, n ≥ 3. Statistical significance is indicated as follows: *p < 0.05; **p < 0.01; ***p < 0.001; n. s., not significant.

DSS, Rabex^HD^, and t-Rabex^LD^ were administered according to the experimental procedures described in Figure 2 and Figure 7. Subsequently, RNA was extracted from the proximal (Figure 7A-D) and distal colons (Figure 7E-H), and gene expression levels were analyzed using qRT-PCR. Drastic increases in all the pro-inflammatory genes tested were observed in both the proximal (Figure 7A) and distal colons (Figure 7E) in the DSS_CTRL groups. Administration of Rabex^HD^ and t-Rabex^LD^ to DSS-treated mice effectively suppressed the expression of these pro-inflammatory genes in both the colons. Additionally, both Rabex^HD^ and t-Rabex^LD^ promoted the expression of anti-inflammatory genes, although no statistical significance was observed for Arg-1 (Figure 7B, F). Relative anti-oxidant (Figure 7C, G) and tight junction maintenance (Figure 7D, H) mRNA levels were increased by the administration of Rabex^HD^ and t-Rabex^LD^ in both regions. It is evident that Rabex^HD^ and t-Rabex^LD^ exert their anti-inflammatory, anti-oxidant, and tight junction maintenance effects, regardless of the colon region. To analyze the inflammatory marker in the entire colon tissue, IL-1β protein levels were measured using ELISA (Figure 7I). The DSS_CTRL group showed an increase in IL-1β protein levels to 550.9 pg/mg, whereas the DSS_Rabex^HD^ and DSS_t-Rabex^LD^ maintained levels at 241.0 pg/mg and 158.5 pg/mg, respectively. This indicates that Rabex and t-Rabex can reduce the expression of DSS-induced cytokine not only in the proximal and distal colon but throughout the entire colon. This ultimately signified that Rabex and t-Rabex were dispersed throughout the colon, thus indicating their potential to obtain a uniform therapeutic effect within the colon. To further substantiate these findings and explore the mechanism of action, additional studies based on the results of GO and KEGG analyses in conjunction with identifying the therapeutic components of Rabex for IBD treatment is required.

To evaluate the biocompatibility of Rabex and t-Rabex, we administered each EV orally to DSS-treated IBD models and collected various organs for analysis (Figure 7J-M). The hemolytic properties of each EV were assessed using collected blood samples (Figure 7J). Given the small size and unique physicochemical properties of nanoparticles, there is a potential for their interaction with red blood cells, ultimately leading to their destruction 73, 74. Although 98.7% red blood cell hemolysis was observed in fully hemolyzed positive CTRL, neither Rabex^HD^ nor t-Rabex^LD^ caused hemolysis of red blood cells, and only 1.0% hemolysis was observed (Figure 7J-L). Additionally, H&E staining was performed on the heart, liver, spleen, lungs, and kidneys, and comparisons were made to the CTRL group (Figure 7M). As a result, we concluded that the administration of Rabex^LD^, Rabex^HD^ and t-Rabex^LD^ did not adversely affect any organ. In summary, Rabex and t-Rabex are expected to reduce concerns regarding potential side effects when applied clinically, as they are highly biocompatible.

Overall, the results confirmed that biocompatible Rabex and t-Rabex operates *in vivo* through a mechanism similar to that observed in the *in vitro* cellular experiments, thus substantiating its efficacy in the DSS-induced colitis model. Notably, at the molecular level, t-Rabex^LD^ despite administration at a dosage ten-fold lower than that of Rabex^HD^, exhibited enhanced therapeutic efficacy due to its targeting functionality.

## Conclusion

The incidence of IBD is increasing globally; however, the cause remains unknown, thus necessitating the development of effective therapies due to its multifaceted and complex mechanisms. In this study, we aimed to develop a novel therapeutic drug for effective treatment of IBD using EVs isolated from red cabbage. The therapeutic efficacy of Rabex was comprehensively assessed using a mouse model of colitis and *in vitro* experiments. The oral administration of Rabex protected the colon from DSS-induced shrinkage, ultimately resulting in a notable reduction in the inflammatory response. Further analysis revealed the dual functionality of Rabex that included suppression of inflammation in macrophages and promotion of colon epithelial cell regeneration that are both crucial for effective IBD treatment. Engineering Rabex with HA to create t-Rabex simultaneously enhanced its ability to target immune and intestinal epithelial cells. *In vitro* and *in vivo* studies examining t-Rabex have demonstrated its superior targeting efficiency and therapeutic efficacy compared to Rabex, thus paving the way for a more effective IBD treatment. Biodistribution studies and molecular profiling confirmed the effective delivery of t-Rabex to the gastrointestinal tract, where it regulated the expression of genes related to its anti-inflammatory, antioxidative, and tight-junction maintenance properties. These findings underscore the potential of Rabex and t-Rabex as precise and effective therapeutic agents for IBD.

Currently, numerous studies focus on plant-derived EVs for the treatment of IBD 75, 76. Compared to these, Rabex and t-Rabex offer several distinct advantages. Firstly, Rabex provides a consistent yield without variation in characterization analyses across multiple batches, ensuring reliability and reproducibility in therapeutic applications. Additionally, Rabex addresses IBD through multiple mechanisms, enhancing its therapeutic potential beyond the single-effect treatments typically seen with other plant-derived EVs. Moreover, t-Rabex, specifically engineered using HA to target the CD44 receptor, offers the distinct advantage of delivering high therapeutic efficacy directly to the inflammatory regions of the colon. This targeted capability allows for effective treatment with lower dosages of Rabex, potentially reducing side effects and increasing treatment precision. However, t-Rabex has some disadvantages compared to naturally sourced Rabex. One drawback is the additional time and cost required for engineering HA onto Rabex. Further studies are needed to evaluate whether the enhanced targeting capabilities justify the time and cost involved in t-Rabex engineering and to explore the potential for optimizing production without compromising efficacy.

## Supplementary Material

Supplementary figures and tables.
