# Supplementary material for: Engineered plant-derived extracellular vesicles for targeted regulation and treatment of colitis-associated inflammation
Source: Theranostics. 2024 Sep 3;14(14):5643–61. doi: 10.7150/thno.97139 (PMC11413791; doi:10.7150/thno.97139)
Supplement: Supplementary file 1 — Supplementary figures and tables. [file thnov14p5643s1.pdf]

Supplementary information

Engineered plant-derived extracellular vesicles for targeted regulation and treatment of colitis-associated inflammation

Su Jin Kang [1], Jeong Hyun Lee [1], Won Jong Rhee [1,2,3,]*

1. Department of Bioengineering and Nano-Bioengineering, Incheon National University Incheon 22012, Republic of Korea.
2. Division of Bioengineering, Incheon National University Incheon 22012, Republic of Korea.
3. Research Center for Bio Materials & Process Development, Incheon National University, Incheon 22012, Republic of Korea

* Corresponding author: Won Jong Rhee, Division of Bioengineering, Incheon National University Incheon 22012, Republic of Korea. Tel: +82-32-835-8299. Email address: wjrhee@inu.ac.kr (W. J. Rhee)

Supplementary Table and Figure

Table S1

Table S1. Disease activity index (DAI) scoring system for DSS-induced colitis

Score	Decrease in growth (%)	Stool consistency	Occult/gross rectal bleeding
0	0	Normal	Normal
1	1 – 5	Normal	Occult blood +
2	5 – 10	Loose stools	Occult blood ++
3	10 – 15	Loose stools	Occult blood +++
4	>15	Diarrhea	Gross bleeding

Table S2

Table S2. Histological scoring system for DSS-induced colitis

Score	Inflammation	Lesions depth	Crypt destruction	Lesions width
0	None	None	None	0
1	Mild	Submucosa	1/3 basal crypt	1 – 25
2	Severe	Muscularis	2/3 basal crypt	26 – 50
3	–	Sera	Intact epithelium only	51 – 75
4	–	–	Total crypt and epithelium	76 – 100

Table S3

Table S3. Primer sequences for qRT-PCR of the mouse genes

Gene	Primer sequence (5' to 3')	
	Forward	Reverse
Actin	CTGAGAGGGAAATCGTGCGT	CCACAGGATTCCATACCCAAGA
IL-1β	CTTCTTTGGGTATTGCTTGGGATC	CCA-GCTTCAAATCTCACAGCAG
IL-6	GACAAAGCCAGAGTCCTTCAGAGA	CACTAGGTTTGCCGAGTAGATCTC
COX-2	GAAGTCTTTGGTCTGGTGCCTG	GTCTGCTGGTTTGGAATAGTTGC
TNF-α	CTGTGAAGGGAATGGGTGTT	GGTCACTGTCCCAGCATCTT
CD206	AGCTTCATCTTCGGGCCTTTG	GGTGACCACTCCTGCTGCTTTAG
Arg-1	AGCTCTGGGAATCTGCATGG	ATGTACACGATGTCTTTGGCAGATA
NQO-1	CATCACAGGTGAGCTGAAGGA	ACAATATCTGGGCTCAGGCG
Nrf-2	GGTTGCCCACATTCCCAAAC	GCAAGCGACTCATGGTCATC
HO-1	AAGCCGAGAATGCTGAGTTCA	GCCGTGTAGATATGGTACAAGGA
ZO-1	CTTCTCTTGCTGGCCCTAAAC	TGGCTTCACTTGAGGTTTCTG
Claudin-1	AGACCTGGATTTGCATCTTGG TG	TGCAACATAGGCAGGACAAGAG
Occludin	CACACTTGCTTGGGACAGAG	TAGCCATAGCCTCCATAGCC

Figure S1

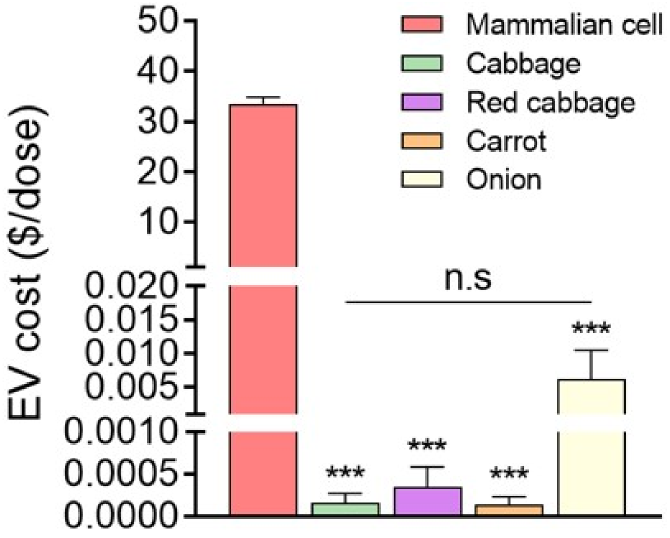

Figure S1. Comparative cost analysis regarding the production of EVs from various sources, including red cabbages. The final EVs cost from 1 dose (5×10^{10} particles) of cabbage (0.0002 $/dose), red cabbage (0.0003 $/dose), carrot (0.0001 $/dose), and onion (0.0062 $/dose), respectively. For plant-derived EVs, the yield was calculated in particles per gram, and the cost per dose was determined based on wholesale prices. In contrast, for mammalian cell-derived EVs, the cost calculation included the price of the culture medium and FBS required for actual EVs production, leading to a price of 33.51 $/dose. The data are presented as mean ± SEM, $n \geq 3$. Statistical significance is indicated as follows: ***$p < 0.001$; n.s, not significant.

Figure S2

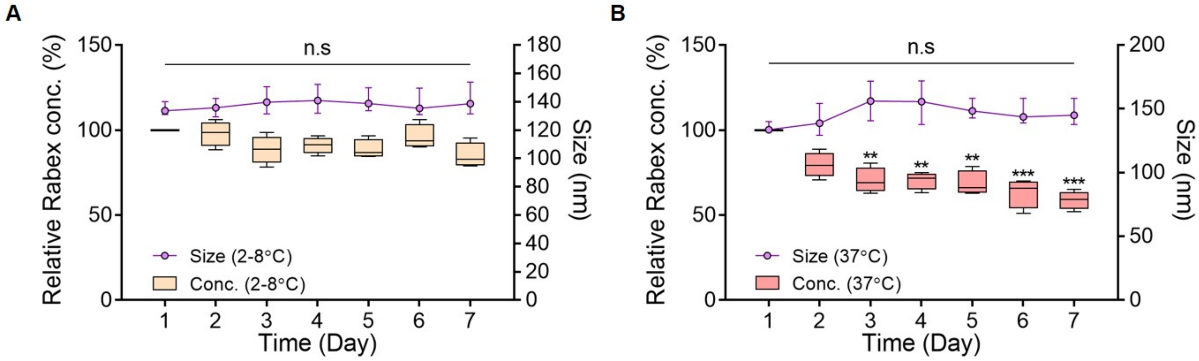

Figure S2. Investigation of Rabex temperature-related stabilities at 2 – 8°C. (**A**) and 37°C (**B**) in 50% serum-containing solution. The sizes and concentrations of Rabex were monitored daily using NTA. The relative Rabex concentration was calculated normalized with respect to the initial concentration. The data are presented as mean ± SEM, n ≥ 3. Statistical significance is indicated as follows: **$p < 0.01$; ***$p < 0.001$; n.s, not significant.

Figure S3

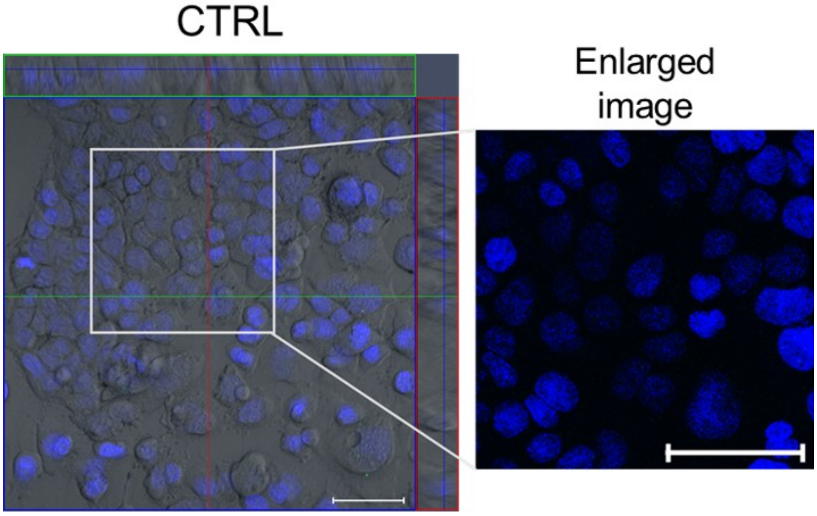

Figure S3. Confocal microscopy analysis of the CTRL group to confirm Rabex intracellular delivery. The analysis was performed under the same conditions as for Rabex using a confocal microscope. Size bars indicate 50 μm.

Figure S4

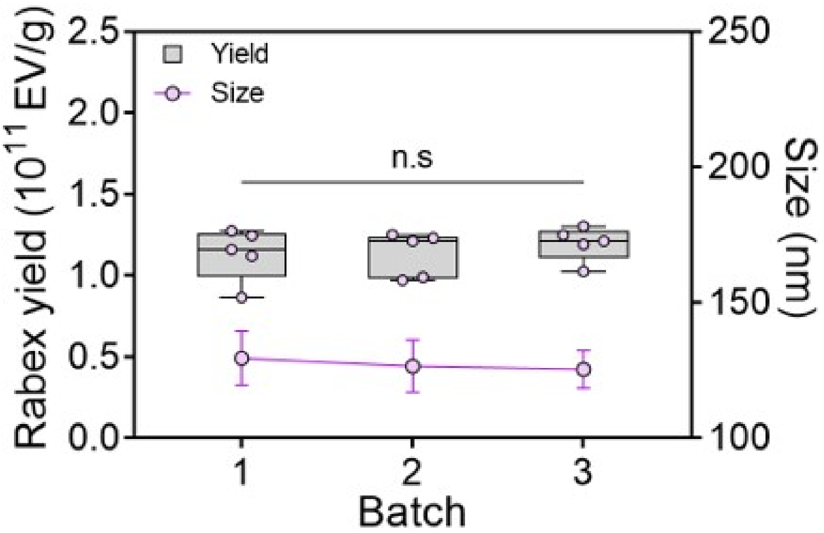

Figure S4. The consistency of Rabex production across various batches. Three batches of Rabex produced from red cabbages over a 3 year period with the identical production process were compared. Rabex yield was shown as the number of Rabex per gram of red cabbages. Note that no significant difference was observed among the batches meaning the consistent Rabex production. The data are presented as mean ± SEM, n ≥ 3. Statistical significance is indicated as follows: n.s, not significant.

Figure S5

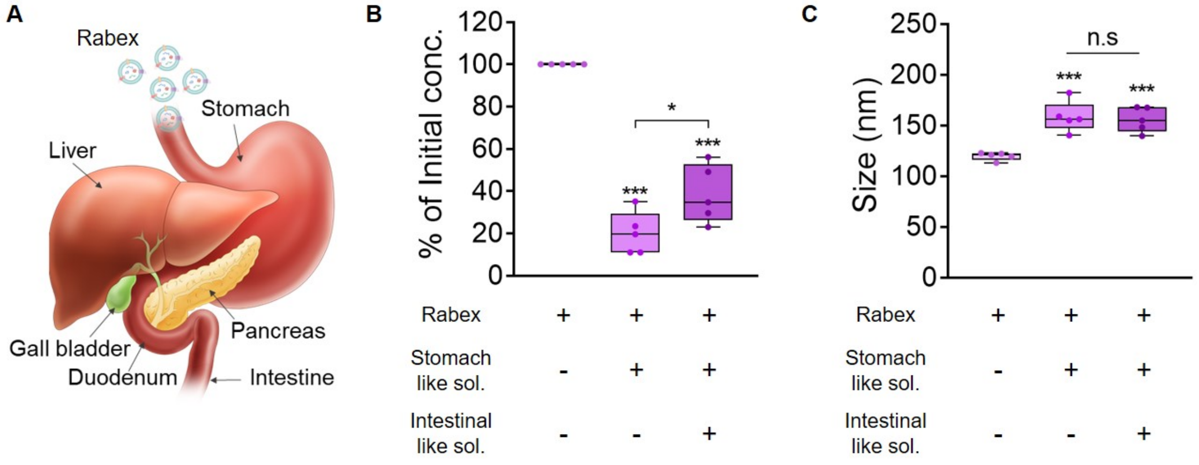

Figure S5. Evaluation of Rabex stability under simulated GI tract conditions. (**A**) An illustrative representation of the various digestive fluids secreted in the GI tract. To mimic the GI environment, solutions analogous to gastric and intestinal fluids were prepared using pH adjustments and enzymes, followed by incubation at 37°C for 1 h. (**B**) Measurement of Rabex concentration post-incubation in each solution using NTA. (**C**) Analysis of the size changes in Rabex under these conditions. This approach provides a comprehensive assessment of Rabex stability in environments replicating the human GI tract. The data are presented as mean ± SEM, $n \geq 3$. Statistical significance is indicated as follows: *$p < 0.05$; ***$p < 0.001$; n.s, not significant.

Figure S6

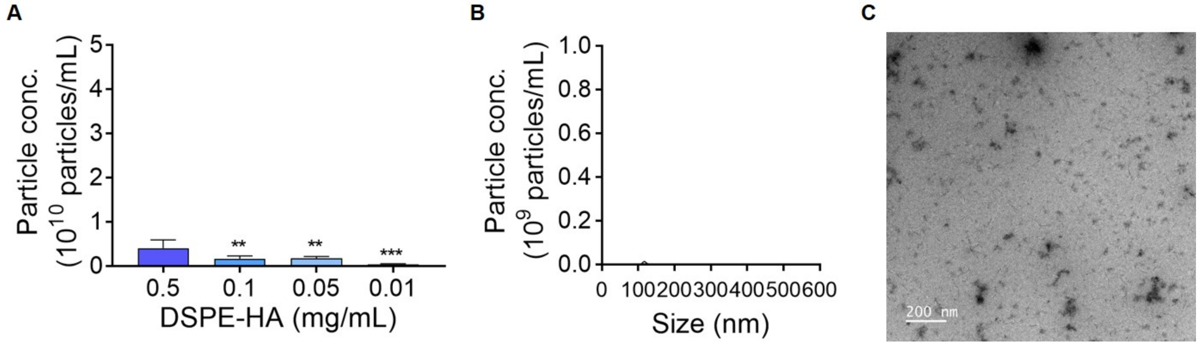

Figure S6. Assessment of micelle formation by DSPE-PEG-HA. To rule out the possibility of unwanted particle formation by DSPE-PEG-HA, various concentrations of DSPE-PEG-HA were incubated and analyzed. (**A**) NTA determining optimal DSPE-PEG-HA concentration to prevent micelle formation. Note that minimal levels of micelle formation were observed for all concentrations, and 0.01 mg/mL of DSPE-PEG-HA was chosen for t-Rabex conjugation. (**B**) Size distribution analysis of DSPE-PEG-HA (0.05 mg/mL) using NTA following a 4 h incubation at 37°C. Note that negligible levels of self-assembled DSPE-PEG-HA particles were detected. (**C**) Morphological assessment through TEM of the same sample post-incubation. Note that negligible number of unwanted particles were formed by DSPE-PEG-HA. The data are presented as mean ± SEM, n ≥ 3. Statistical significance is indicated as follows: $^{**}p < 0.01$; $^{***}p < 0.001$.

Figure S7

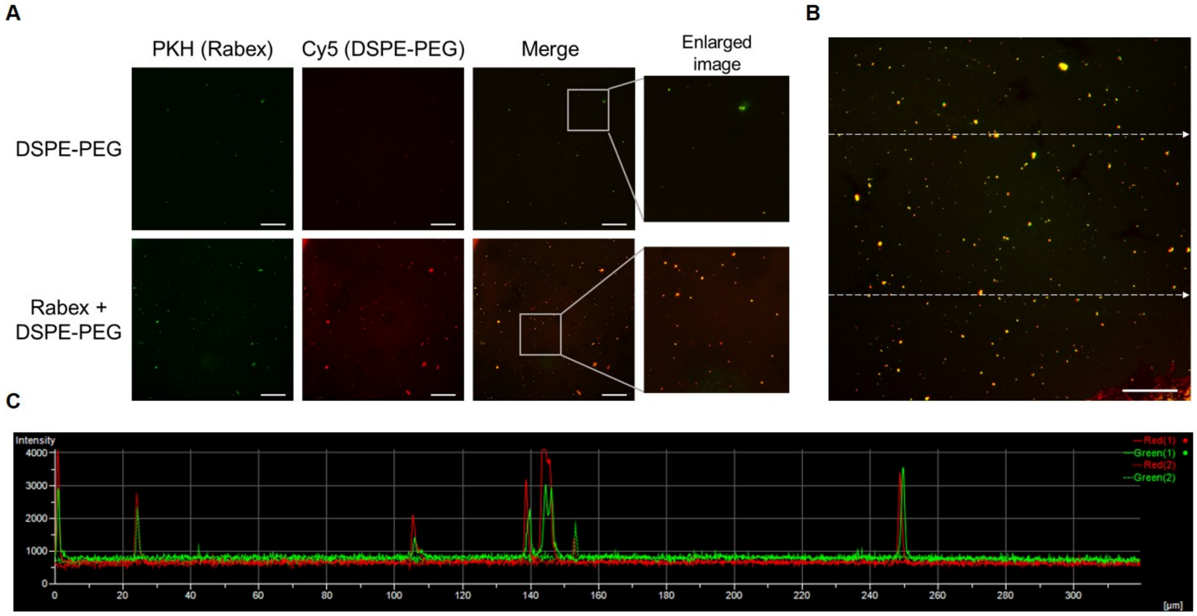

Figure S7. Fluorescence microscopy for the confirmation of t-Rabex construction. To verify DSPE-PEG-HA was inserted and conjugated on the surface of Rabex, Cy5-labeled DSPE-PEG (Red) was incubated with PKH stained Rabex (Green), and further incubated for 4 h at 37°C. (**A**) Co-localization of Rabex and DSPE-PEG were observed using fluorescent microscope. The enlarged images show the presence of Rabex and DSPE-PEG at the same positions. (**B**-**C**) Peak analysis from fluorescent microscopy showing Rabex and DSPE-PEG co-localization. (**B**) Two dot lines were randomly chosen from the representative image of PKH-labeled Rabex and Cy5-labeled DSPE-PEG conjugation. (**C**) The fluorescent dots on the two lines were further analyzed to verify the overlapping of peaks from each fluorescent dye. Note that the most of observed peaks exhibit overlapping green and red fluorescence across the positions.

Figure S8

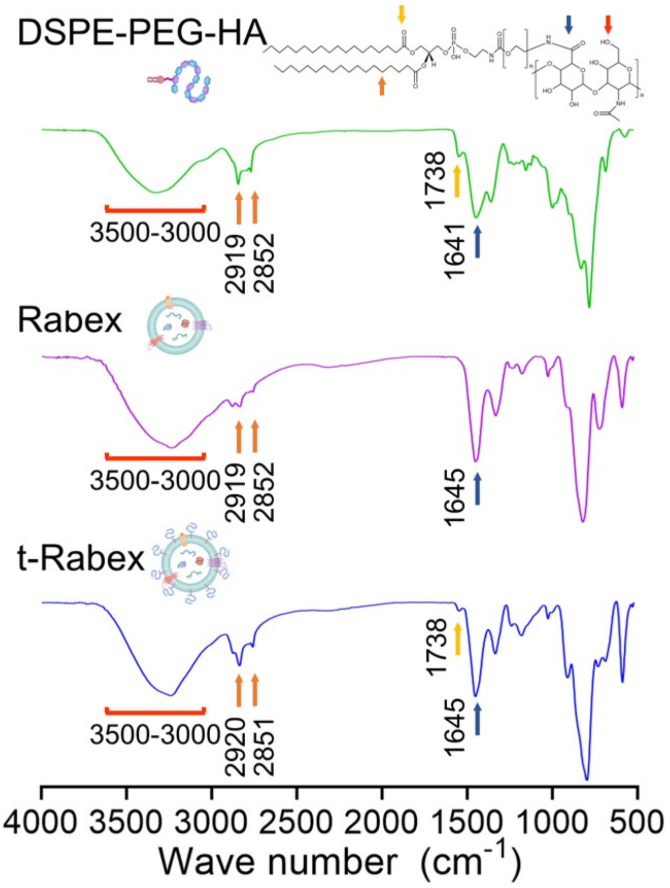

Figure S8. FT-IR spectra of DSPE-PEG-HA, Rabex, and t-Rabex. Conjugation was confirmed using FTIR spectroscopy. Yellow arrows (1,738 cm^{-1}) indicate the carbonyl groups of DSPE-PEG-HA.

Figure S9

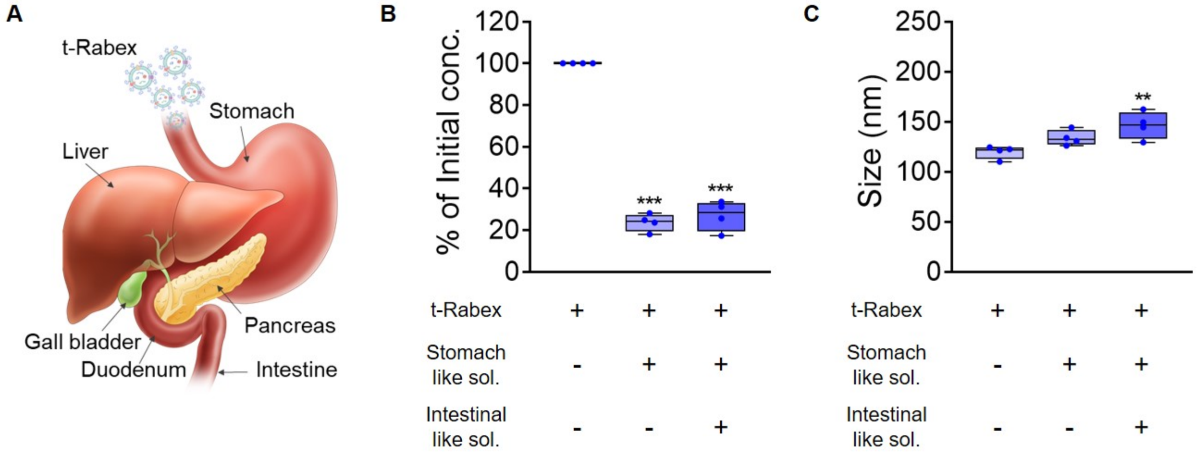

Figure S9. **Evaluation of t-Rabex stability under simulated GI tract conditions.** (**A**) An illustrative representation of the various digestive fluids secreted in the GI tract. The experiment was conducted under the same conditions as Figure S5. (**B**) Measurement of Rabex concentration post-incubation in each solution using NTA. (**C**) Analysis of the size changes in Rabex under these conditions. The data are presented as mean ± SEM, $n \geq 3$. Statistical significance is indicated as follows: $^{**}p < 0.01$; $^{***}p < 0.001$.

Figure S10

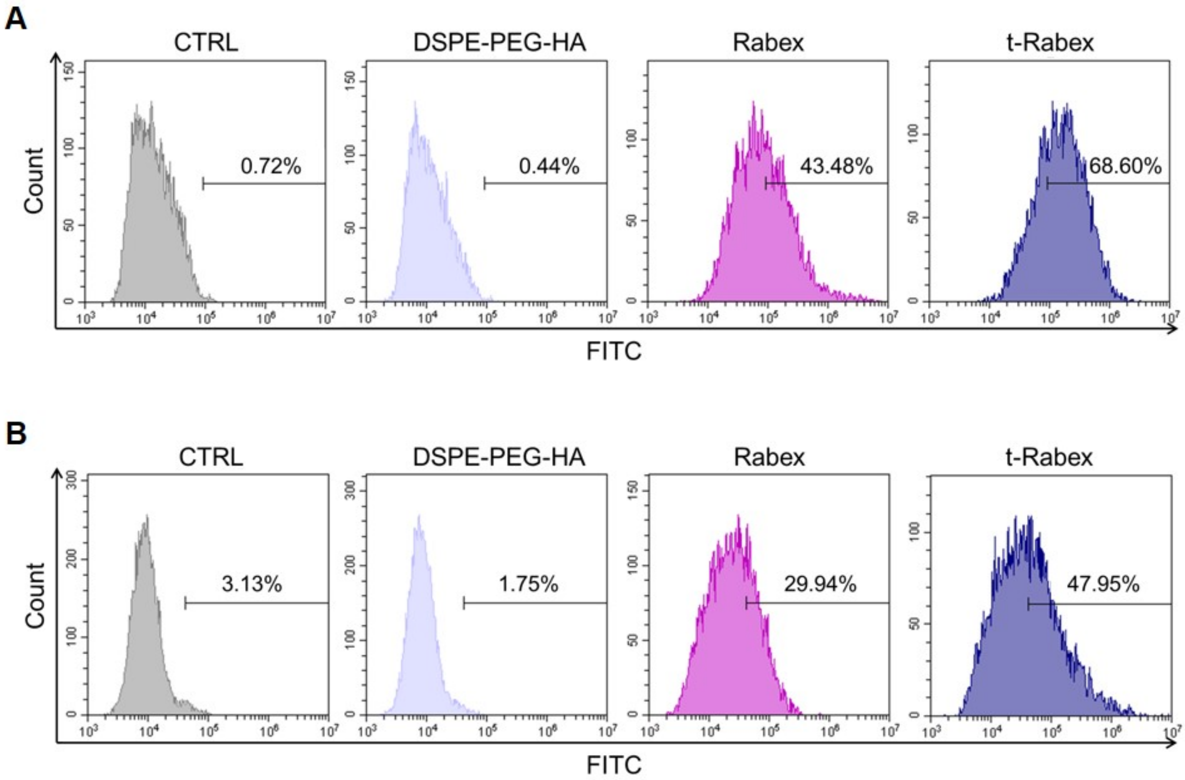

Figure S10. Assessment of t-Rabex targeting ability in epithelial cells and macrophages. (**A**) Flow cytometric analysis demonstrating enhanced t-Rabex delivery to Caco-2 cells. Rabex and t-Rabex were stained with PKH staining dye, respectively, and DSPE-PEG-HA without Rabex was used as negative control. Note that higher amount of t-Rabex was uptaken by colon epithelial cells as compared to Rabex. (**B**) Flow cytometric analysis of Rabex and t-Rabex uptake in THP-1 cells. Note that the enhanced delivery effect of t-Rabex is not confined to colon epithelial cells but also extents to macrophages.

Figure S11

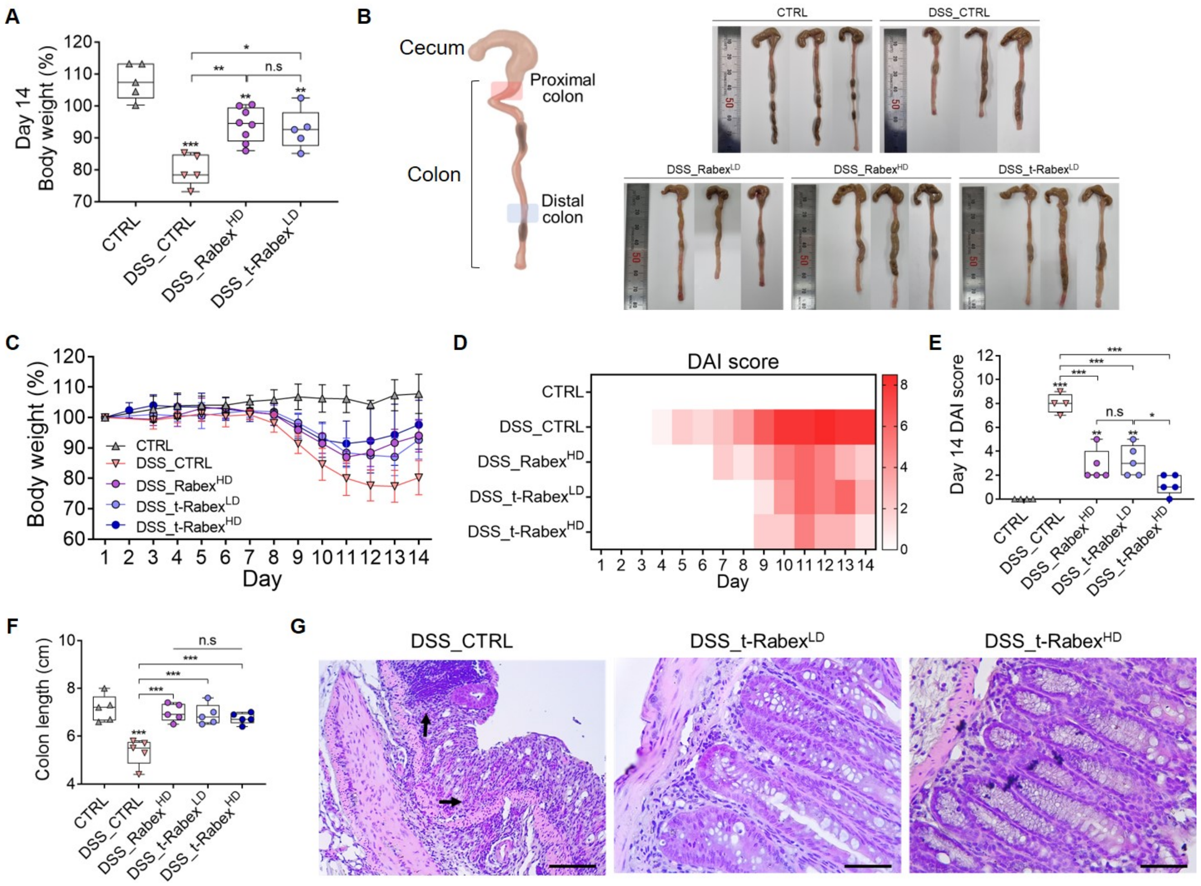

Figure S11. Investigating the therapeutic efficacy of t-Rabex in an IBD model. (**A**) The comparison of weight reduction on day 14 in both CTRL, DSS_CTRL, DSS_ $Rabex^{HD}$, and DSS_t-$Rabex^{LD}$ administered groups. (**B**) Schematic representation of proximal and distal colon sections and images showing colon length changes induced by DSS treatment. (**C-G**) Results of evaluating the therapeutic effect of t-Rabex at a high dose (t-$Rabex^{HD}$) on IBD. t-$Rabex^{HD}$ was administered orally at a concentration of 5×10^{11} particles/mL for 10 doses. (**C**) Body weight loss, (**D**, **E**) changes in DAI score, and (**F**) colon length induced by DSS treatment were measured on day 14 in t-$Rabex^{HD}$ administered groups. (**G**) H&E staining of colon tissues in inflamed regions (black arrow), with scale bars indicating 50 µm. The data are presented as mean ± SEM, n ≥ 5. Statistical significance is indicated as follows: *p < 0.05; **p < 0.01; ***p < 0.001; n.s, not significant.

Figure S12

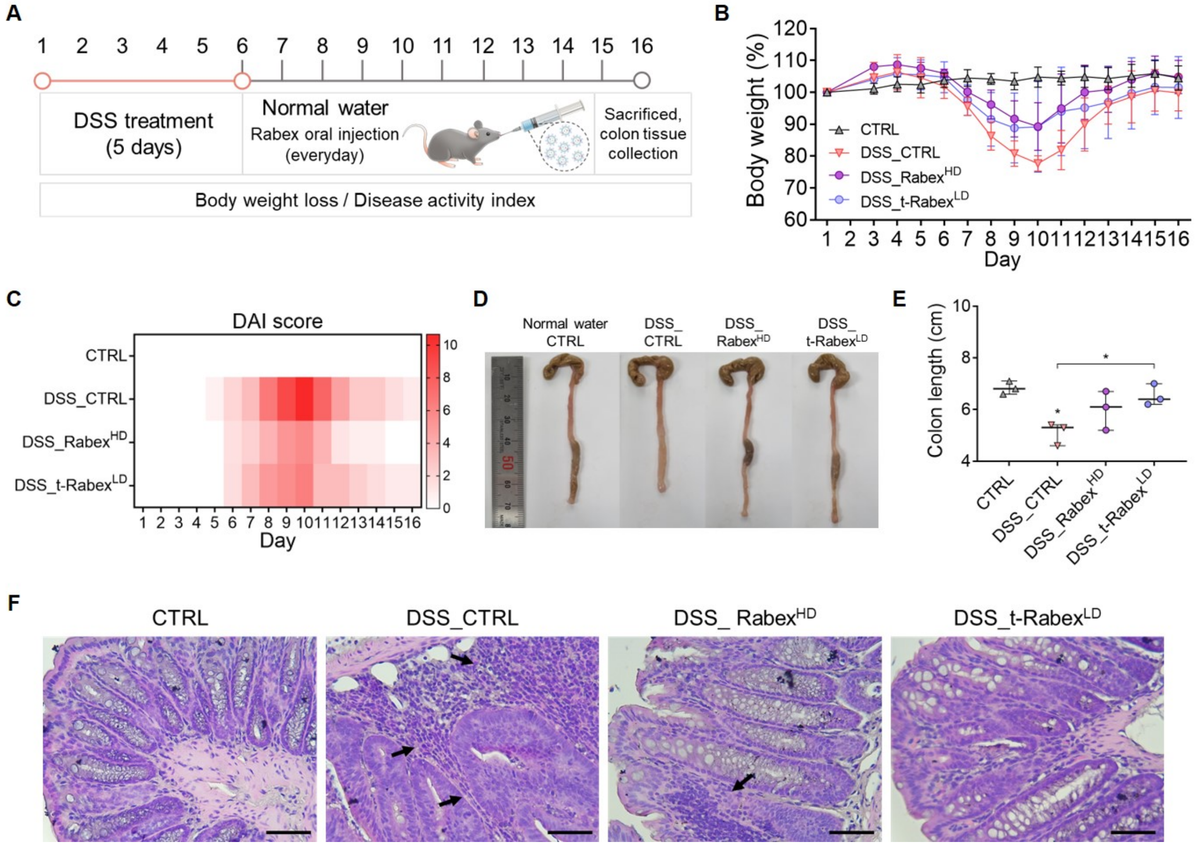

Figure S12. Evaluation of therapeutic efficacy of Rabex^{HD} and t-Rabex^{LD} post DSS induction. (**A**) After administering DSS for 5 days, Rabex^{HD} and t-Rabex^{LD} were orally administered for a total of 10 doses to evaluate their effects. (**B**) Body weight loss on day 16, (**C**) changes in DAI score, and (**D**, **E**) colon length measured in DSS-treated groups. (**F**) H&E staining of mouse colon tissues showed a higher degree of tissue recovery in the DSS_Rabex^{HD} and DSS_t-Rabex^{LD} groups, with scale bars indicating 50 μm. The data are presented as mean ± SEM, n ≥ 3. Statistical significance is indicated as follows: *$p < 0.05$; n.s, not significant.

Figure S13

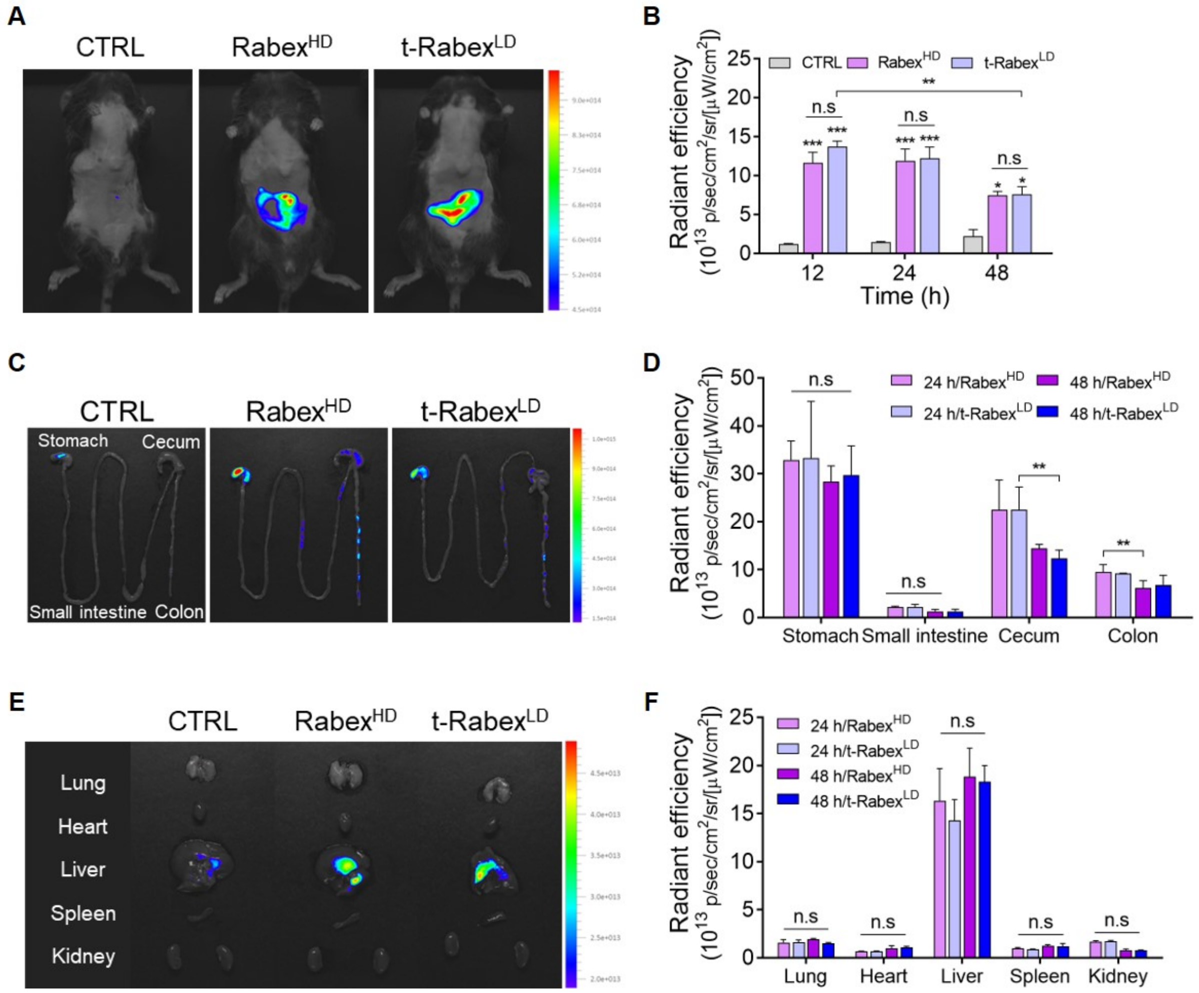

Figure S13. *In vivo* biodistribution of t-Rabex. Fluorescence imaging and analysis 48 h post-administration using IVIS system. (**A**, **B**) Fluorescence imaging of the ventral area (left images) and quantitative analysis indicating radiant efficiency of Rabex^{HD} and t-Rabex^{LD} (right graph). (**C**, **D**) *In vivo* tracing across the GI tract and quantitative analysis indicating radiant efficiency for each section (stomach, small intestine, cecum, and colon). (**E**, **F**) Fluorescent imaging and the resulting radiant efficiency in major organs (lungs, heart, liver, spleen, and kidneys). The data are presented as mean ± SEM, $n \geq 3$. Statistical significance is indicated as follows: *$p < 0.05$; **$p < 0.01$; ***$p < 0.001$; n.s, not significant.
